# Piezo1-mitochondrial calcium uniporter coupling-dependent mechanotransduction basis for acupuncture to drive analgesia

**DOI:** 10.1186/s13020-026-01502-0

**Published:** 2026-10-28

**Authors:** Kaifang Yao, Jiaqi Xia, Baomin Dou, Zezhi Fan, Shamim Md. Forhad, Zhihan Chen, Li Ma, Junyi Li, Kaiyuan Deng, Xiaowei Lin, Puyu Niu, Yuxing Fang, Yangyang Liu, Shenjun Wang, Zhifang Xu, Yi Guo

**Affiliations:** 1 Research Center of Experimental Acupuncture Science, School of Acupuncture & Moxibustion and Tuina, Tianjin University of Traditional Chinese Medicine, Tianjin, 301617 People’s Republic of China; 2 Tianjin Key Laboratory of Modern Chinese Medicine Theory of Innovation and Application, School of Traditional Chinese Medicine, Tianjin University of Traditional Chinese Medicine, Tianjin, 301617 China; 3 National Clinical Research Center for Chinese Medicine, Tianjin, 301617 People’s Republic of China; 4 School of Chinese Medicine, Tianjin University of Traditional Chinese Medicine, Tianjin, 301617 People’s Republic of China

**Keywords:** Acupuncture analgesia, Piezo1, Inflammatory pain, Calcium signaling, Sarcoplasmic reticulum-mitochondria axis, Adenosine

## Abstract

**Background:**

The mechanosensitive ion channel Piezo1 is a critical sensor of mechanical forces in diverse biological contexts, albeit its role in translating the acupuncture-induced mechanical stimulation into analgesic signaling remains largely unexplored. Acupuncture is a widely used therapy for pain management, with needle manipulation-generated mechanical forces considered central to its therapeutic efficacy. However, the molecular mechanisms underlying this mechanotransduction process remain unknown. Here, we identified myocyte Piezo1 at the ST36 acupoint as a key initiator converting local needling force into peripherally derived analgesic signaling under complete Freund’s adjuvant (CFA)-induced inflammatory pain conditions.

**Methods:**

In mice with CFA-induced inflammatory pain, we assessed the analgesic efficacy of deep needling at ST36 using paw withdrawal latency tests. The underlying molecular mechanisms were examined through immunohistochemistry, immunofluorescence, western blotting, transmission electron microscopy, enzyme-linked immunosorbent assay, electromyography, and in vivo two-photon calcium (Ca) imaging. To evaluate the Piezo1-Ca^2^⁺-calmodulin (CaM)-Ca^2^⁺/CaM-dependent protein kinase II (CaMKII)-signaling axis and its downstream effects on mitochondrial function and purinergic signaling, we employed muscle-specific adeno-associated virus-mediated knockdown of Piezo1 and CaM together with a panel of pharmacological modulators (GsMTx4, Yoda1, TFP, 2-APB, RU360, DPCPX).

**Results:**

We first confirmed Piezo1 expression in myocytes within the ST36 acupoint, and muscle-specific Piezo1 knockdown abrogated the analgesic effects of deep needling at ST36. Mechanical stimulation by needling activated Piezo1, thereby triggering Ca^2^⁺ influx, increasing CaM expression and CaMKII phosphorylation, and enhancing sarcoplasmic reticulum-mitochondrial coupling via the inositol trisphosphate receptor-1 (IP3R1)-glucose-regulated protein 75 (GRP75)-voltage-dependent anion channel-1 (VDAC1) complex and mitochondrial Ca uniporter (MCU) pathway. These events promoted mitochondrial Ca^2^⁺ uptake and increased adenosine triphosphate (ATP)/adenosine production. Both genetic and pharmacological inhibition of Piezo1, CaM, or downstream signaling molecules blocked the analgesic effects of acupuncture, whereas direct Piezo1 activation with Yoda1 mimicked these effects. In vivo two-photon Ca imaging confirmed that electroacupuncture-evoked myocyte Ca^2^⁺ transients are Piezo1-dependent, and the resulting adenosine may mediate remote analgesia through the activation of the adenosine A1 receptor.

**Conclusion:**

Piezo1 acts as a core peripheral mechanotransducer that converts local mechanical stimuli generated by acupuncture into downstream analgesic signaling in CFA-induced inflammatory pain. This study delineates a specific myocyte-originated mechanotransduction axis underlying acupuncture analgesia under inflammatory conditions, thereby providing mechanistic insights for understanding acupoint-specific therapeutic effects.

**Supplementary Information:**

The online version contains supplementary material available at https://doi.org/10.1186/s13020-026-01502-0.

## Introduction

Pain is an unpleasant sensory and emotional experience associated with tissue damage or inflammation. It often involves persistent central and peripheral sensitization, amplified neuroinflammation, and neural circuit remodeling [1]. Although non-steroidal anti-inflammatory drugs and opioids remain the mainstay of clinical analgesia, their long-term use is limited by gastrointestinal injury, drug dependence, and side effects on the central nervous system (CNS), which render them suboptimal for the management of chronic pain and neuroinflammatory conditions that require sustained treatment [2]. In this context, somatic sensory stimulation therapies have gained increasing attention. Acupuncture, a representative non-pharmacological intervention, has been integrated into the World Health Organization’s traditional medicine strategy and is now widely used for pain management. High-quality evidence supports its efficacy in inflammatory conditions such as rheumatoid arthritis [3–7].

Understanding how specific sites of the body surface perceive external stimuli and transmit signals to the CNS, thereby integrating autonomic outflow to exert systemic effects, has become a central focus of somatosensory–autonomic reflex biology. Mechanical stimulation at acupoints activates local sensory receptors, converting physical forces into electrochemical signals that trigger peripheral-to-central analgesic and anti-inflammatory pathways. For instance, electroacupuncture (EA) at ST36 activates the sciatic nerve and vagal–adrenal axis to trigger catecholamine release and control inflammation [8]. Landmark studies have further demonstrated that PROKR2-expressing sensory neurons, characterized by their distinctive distribution in the deep hindlimb fascia, provide the neuroanatomical basis for acupoint-specific vagal–adrenal anti-inflammatory responses [9]. Clinical evidence indicates that manual acupuncture techniques, such as twisting and lifting, improve therapeutic outcomes in conditions including migraine, underscoring the critical role of mechanical force in acupoint activation [10]. During this process, neurogenic inflammation releases substance P, which sensitizes afferent nerves and amplifies acupuncture-induced signals [11]. Furthermore, mechanical stimulation triggers ATP release from acupoint sites, leading to local adenosine (ADO) accumulation and subsequent activation of sensory nerve terminals via A1 receptors to produce analgesic effects [12].

Piezo1 is a highly sensitive mechanosensitive Ca^2^⁺-permeable channel that responds to mechanical stretch within milliseconds and mediates extracellular Ca^2^⁺ entry, thereby activating downstream signaling pathways, including protein kinase C (PKC) and CaMKII [13–15]. Piezo1 activation can induce cytoplasmic calcium (Ca) oscillations and CaMKII phosphorylation, regulating diverse biological processes such as intestinal barrier integrity and cytokine secretion [16, 17]. Notably, Piezo1 is highly expressed in mechanosensitive tissues, including skeletal and smooth muscles, while the ST36 acupoint area primarily consists of muscle tissue as its structural basis. This histological distribution characteristic provides crucial histological support for Piezo1-mediated detection of acupuncture-generated mechanical stimuli [18]. Although our previous studies have implicated transient receptor potential channels, such as Transient Receptor Potential Vanilloid 1 (TRPV1) and Transient Receptor Potential Ankyrin 1 (TRPA1), in acupuncture-induced analgesia, these channels exhibit limited sensitivity to mechanical stimuli [19, 20]. Whether Piezo1 serves as the primary mechanosensor that initiates local Ca^2^⁺ signaling at acupoints in response to acupuncture-generated force remains to be systematically elucidated.

In this study, we tested the hypothesis that Piezo1 serves as a local mechanoreceptor at the ST36 acupoint, converting acupuncture-induced mechanical stimulation into Ca^2^⁺/CaM/CaMKII signaling. We further explored how this signaling axis modulates sarcoplasmic reticulum (SR)–mitochondrial Ca transfer and cellular energy metabolism to promote ADO production, ultimately alleviating neuroinflammatory pain. By elucidating this mechanotransduction pathway linking acupoint stimulation to analgesic outcomes, our study aims to provide new mechanistic insights into acupuncture-induced analgesia in complete Freund’s adjuvant (CFA)-triggered inflammatory pain. This work provides critical experimental evidence for the biological mechanism underlying acupuncture-induced acupoint stimulation and therapeutic effects and establishes a theoretical foundation for the development of analgesic strategies targeting the Piezo1 pathway.

## Materials and methods

### Experimental animals

This study utilized specific pathogen-free male C57BL/6 J mice (age 7–8 weeks, weight 20–22 g) purchased from Beijing Vital River Laboratory Animal Technology Co., Ltd. (License No.: SCXK (Jing) 2021-0006). All experimental procedures were strictly conducted in accordance with the Guide for the Care and Use of Laboratory Animals and were approved by the Laboratory Animal Ethics Committee of Tianjin University of Traditional Chinese Medicine (Approval No.: TCM-LAEC2021279; Usage License No.: SYXK (Jin) 2019-0004). Before the experiment, the animals were acclimatized for 1 week under standard conditions (temperature: 25 ± 2 °C, humidity: 55% ± 5%, natural light cycle) with 6 mice per cage, allowed free access to food and water, and housed in a well-ventilated and clean environment. All experimental mice were randomly assigned to different experimental groups using a random number table before modeling to eliminate subjective grouping bias.

### CFA-induced inflammatory pain model

The CFA-induced inflammatory pain model was established using a standard protocol. The mice were properly restrained, and the skin of their right hind paw was disinfected. The model group received an intradermal injection of 0.05 mL CFA suspension (Sigma, F5881-10 mL) into the right hind paw. Then, sterile dry cotton balls were gently pressed on the needle hole for 30–60 s to prevent leakage. Control (Con) mice were injected with an equal volume (0.05 mL) of sterile 0.9% saline in the same manner. At 24 h after modeling, successful model induction was objectively confirmed by observing significant paw swelling and redness, along with a significant reduction in thermal pain latency as detected by the radiant heat pain threshold test.

### Treatment intervention

A single acupuncture intervention was administered on the first day after the successful establishment of the model. Bilateral ST36 acupoints were stimulated in the mice. Manual acupuncture (MA) group: Disposable sterile acupuncture needles (0.16 mm × 7 mm) were inserted perpendicularly to a depth of approximately 2–3 mm. After the operator achieved the characteristic "heavy and tight" sensation (Deqi), the reinforcing-reducing method was applied. In this method, the needle was rotated evenly at 180 rpm for 2 min. This manipulation was repeated every 5 min for four cycles [21]. The total needle retention and treatment time were 30 min. Shallow acupuncture group (SACU): The same needle type was used to perform subcutaneous shallow insertion at the same acupoints to a depth of approximately 1 mm, without pursuing Deqi. The subsequent manipulation techniques, operation frequency, needle retention sequence, and total treatment duration were consistent with those used in the acupuncture group. In preliminary experiments, this depth (1 mm) was confirmed to be confined to the subcutaneous layer without the needle penetrating the underlying muscle fascia, as verified through anatomical dissection. EA group: Electrical stimulation (1 mA, 1 Hz dense-disperse wave) was delivered via electrodes connected to ST36 (anode) and ST25 (cathode). ST36 is innervated by lumbar spinal segments that correspond to the afferent input of the inflamed hind paw, whereas ST25 is associated with thoracolumbar spinal segments that do not overlap with the afferent innervation of the hindlimb. Hence, ST25 was selected as the control paired acupoint for EA in this experiment. Ca signal recording was divided into baseline (1 min), stimulation (2 min), and needle retention (2 min) phases. All manual acupuncture manipulation parameters (including insertion depth, rotation frequency, manipulation interval, and total retention time) were strictly standardized according to a unified operating protocol to ensure consistent mechanical stimulation intensity across individual animals.

### Measurement of nociceptive thresholds

Thermal pain threshold was measured using the BME-410C thermal radiation analgesia meter. The mice were acclimated to the test environment for 30 min before measurement. The radiant heat source was focused on the plantar surface of the right hind paw, and the PWL was automatically recorded. Measurements were taken at 5‑min intervals for three consecutive trials, and the average value was calculated. Assessments were performed before modeling, 24 h after modeling, and 30 min after the intervention. A cutoff time of 30 s was applied to avoid tissue damage.

### Sample collection

All tissue samples were collected on day 1 after modeling, following the completion of behavioral tests. For morphological examination, the mice were anesthetized by administering an intraperitoneal injection of tribromoethanol (Aibei, M2910). The dosage was 0.4–0.6 mL per mouse based on body weight, administered slowly at a rate of 10 s/0.1 mL. Upon loss of corneal reflex and muscle relaxation, transcardial perfusion was performed. Approximately 20 mL of ice‑cold saline was perfused rapidly until the liver and limbs became pale. Subsequently, 20 mL of freshly prepared 4% paraformaldehyde (Solarbio, P1110; for immunohistochemistry (IHC)/immunofluorescence (IF)) or 4% glutaraldehyde (Servicebio, G1102; for transmission electron microscopy (TEM)) was perfused until the body became rigid. Bilateral ST36 acupoint tissues were then harvested, consisting of skin and subcutaneous muscle tissue blocks centered on the acupoint (approximately 2 mm × 2 mm, extending 2–3 mm deep), with care taken to minimize mechanical damage such as traction and compression during the procedure. To obtain samples for TEM, tissue blocks were trimmed into 1‑mm^3^ cubes within 1 min, placed in a dish containing fresh glutaraldehyde fixative, transferred to a microcentrifuge tube with fresh fixative, and stored at 4 °C. To obtain samples for protein detection, the mice were sacrificed by cervical dislocation. Bilateral ST36 muscle‑layer tissues were rapidly dissected, placed in pre‑cooled cryotubes, flash‑frozen in liquid nitrogen, and stored at − 80 °C for subsequent western blotting (WB) and enzyme-linked immunosorbent assay (ELISA).

### Western blotting

Protein expression in tissues from the ST36 acupoint region was assessed through WB. Approximately 50 mg of frozen tissue was homogenized on ice in RIPA lysis buffer (Solarbio, R0010) supplemented with 1% PMSF (Solarbio, P0100) and 1% phosphatase inhibitor and lysed for 1.5 h at 4 °C. After the lysate was centrifuged at 12,000 × *g* for 15 min, the supernatant was collected. Protein concentration in the supernatant was measured using a BCA kit (Solarbio, AR0146), and all samples were normalized to 2.5 μg/μL and denatured at 100 °C. Equal amounts of protein (25 μg per lane) were separated on 4–12% Bis–Tris precast gels (GenScript, M41215C) through SDS-PAGE at 140 V constant voltage. Proteins were subsequently transferred to PVDF membranes using wet transfer (100 V, 90 min). After the transfer, the membranes were blocked with protein-free rapid blocking buffer (Solarbio, AR0041) and incubated with the following primary antibodies at 4 °C overnight: anti-p-CaMKII (Thr286) (Cell Signaling Technology, 12716 T, 1:1000), anti-CaMKII (Cell Signaling Technology, 4436 T, 1:1000), anti-Piezo1 (Novus, NBP1-78446SS, 1:100), anti-CaM (Abcam, ab45689, 1:1000), anti-p-IP3R (Ser1756) (Cell Signaling Technology, 8548 T, 1:100), anti-GRP75 (Abcam, ab227215, 1:1000), anti-VDAC1 (Boster, A01168-2, 1:1000), anti-p-MCU (Ser92) (Novus, NBP3-27,827, 1:1000). GAPDH (Boster, A00227-1, 1:10,000) and COX IV (Boster, M05442-1, 1:2000) were used as loading controls. After the membranes were extensively washed with TBST (Solarbio, T1082), they were incubated with HRP-conjugated goat anti-rabbit secondary antibody (SAB, L3032, 1:10,000) for 1 h at room temperature. Following a final wash, protein bands were visualized using an ECL substrate (Glpbio, GK10008). Band intensities were quantified using ImageJ software, and relative protein expression was calculated as the ratio of the target protein grayscale value to that of the corresponding loading control. Each experiment was independently repeated at least three times.

### Immunohistochemical staining

CaM and Piezo1 expression in acupoint tissues was detected through IHC. After dewaxing, hydration, high-pressure heat-induced antigen retrieval in EDTA buffer (pH 9.0; ZY‑1009), and blocking of endogenous peroxidase with 3% H₂O₂, paraffin sections were blocked with 5% BSA (Solarbio, SW3015-100 ml) and incubated overnight at 4 °C with rabbit anti-CaM antibody (Abcam, ab45689, 1:25) or rabbit anti-Piezo1 antibody (Novus, NBP1-78446SS, 1:25). After the sections were washed with PBS and rewarmed at 37 °C the next day, the reaction enhancer and enhanced HRP polymer secondary antibody were sequentially added dropwise for incubation, followed by DAB color development and hematoxylin counterstaining. The samples were then dehydrated through a graded ethanol series, cleared with xylene, and mounted with neutral balsam (Zhongshan Golden Bridge, ZLI-9555). The treated samples were observed, and image acquisition was performed under an optical microscope. The average optical density value of CaM-positive areas was calculated using ImageJ software to quantify the CaM expression.

### IF staining

CaM expression and localization in acupoint tissues were detected through IF staining. Paraffin sections were deparaffinized with xylene and rehydrated through a graded ethanol series, followed by microwave-mediated antigen retrieval using EDTA buffer. After the sections were washed with PBS, tissue areas were circled with an immunohistochemical pen and blocked with a serum blocking solution at room temperature for 30 min. Subsequently, rabbit anti-CaM primary antibody (Abcam, ab45689) diluted 1:25 in antibody diluent was added to the sections and incubated overnight in a humid chamber at 4 °C. The next day, after the sections were thoroughly washed with PBS, DyLight 594-labeled goat anti-rabbit IgG secondary antibody (Boster, BA1127, 1:200) was added to the sections and incubated at room temperature for 50 min in the dark. After the sections were washed with PBS, DAPI staining solution (Solarbio, C0065) was applied to counterstain the nuclei for 2 min at room temperature. Finally, the sections were mounted with an anti-fade mounting medium (Southern Biotech, 0100-20). When the sections were observed under a fluorescence microscope, CaM-positive signals appeared as red fluorescence and nuclei as blue. For each sample, three non-overlapping fields were randomly selected, and the average fluorescence intensity of CaM-positive areas was calculated using ImageJ software to quantify the CaM expression.

### IF double-labeling staining

Double IF staining was performed to determine the cellular localization of CaM and Piezo1 in acupoint tissues. After dewaxing, antigen retrieval (EDTA buffer), and blocking, the sections were incubated with mixed primary antibodies in a humidified chamber at 4 °C overnight. The primary antibody combinations used were as follows: rabbit anti-CaM antibody (Abcam, ab45689, 1:25) mixed respectively with mouse anti-Tryptase (Santa, Sc-59587, 1:200), anti-Vimentin (Santa, Sc-373717, 1:200), anti-CD68 (Santa, Sc-20060, 1:200), or anti-Myosin antibody (Thermo Fisher, 53-6503-80, 1:200); rabbit anti-Piezo1 antibody (Novus, NBP1-78446SS, 1:25) mixed respectively with the aforementioned mouse antibodies or anti-CD31 antibody (NB, A23753, 1:200). The next day, after the sections were washed with PBS, the corresponding fluorescent secondary antibodies were added: Alexa Fluor 594-labeled goat anti-rabbit IgG (Boster, BA1127, 1:200) and Alexa Fluor 488-labeled goat anti-mouse IgG (1:200). Nuclei were counterstained with DAPI, and slides were mounted with antifade medium. Images were acquired using the fluorescence microscope, and colocalization was analyzed using ImageJ software. Regions of interest corresponding to each channel were defined, and the number of double‑positive cells in overlapping areas was quantified to assess the expression and distribution of CaM and Piezo1 in mast cells, fibroblasts, macrophages, endothelial cells, and myocytes.

### Virus preparation and injection at ST36 acupoint

This study utilized serotype AAV2/MyoAAV 2A recombinant adeno-associated viruses (AAVs) provided by Wuhan Shumi Biotechnology Co., Ltd., including the following three categories: (1) myocyte-specific calmodulin knockdown virus rAAV-MHCK7-EGFP-5′miR-30a-shRNA2 (Calm1)-3′-miR30a-WPREs and its negative control virus rAAV-MHCK7-EGFP-5′miR-30a-shRNA(scramble)-3′-miR30a-WPREs 3′iR3Da WPREs (titer: 5.45 × 10^12^ vg/mL); (2) myocyte Piezo1 knockdown virus rAAV-MHCK7-EGFP-5′miR-30a-shRNA(piezo1)-3′-miR30a-WPREs and its negative control virus rAAV-MHCK7-EGFP-5′miR-30a-shRNA(scramble)-3′-miR30a-WPREs (titer: 6.03 × 10^12^ vg/mL); and (3) Ca imaging virus rAAV-MHCK7- GCaMP6m-WPRE and control virus rAAV-MHCK7-EGFP-WPRE (both titer: 4.95 × 10^12^ vg/mL). During viral injection, the bilateral ST36 acupoint regions in the mice were accurately located. Using a microsyringe, 20 μL of the corresponding viral suspension was slowly injected into the muscle layer of the acupoint region. After injection, the needle was retained for 30 s before slowly withdrawing it to prevent viral leakage. Twenty-one days after the administration of all viral injections, tissue samples were collected from the Zusanli acupoint region to prepare frozen sections. The expression of EGFP or GCaMP6m fluorescence was observed using the fluorescence microscope to verify successful viral transduction in the target tissue.

### Drug preparation and injection at ST36 acupoint

Multiple classes of small-molecule drugs and peptides were used for local intervention at the ST36 acupoint site to regulate specific signaling pathways. CaM and Ca signaling pathway modulators: The CaM-specific antagonist trifluoperazine (TFP, GLPBIO, GC63240-5) was prepared as a 5 mg/mL stock solution in DMSO and used at working concentrations of 25 µmol/L (low dose) and 50 µmol/L (high dose), with an equal volume of 1% DMSO as solvent control. The intracellular Ca chelator BAPTA-AM (GLPBIO, GC13517-5) was prepared in physiological saline containing 1% DMSO and used at working concentrations of 25 and 50 µmol/L. The Ca ionophore A23187 (GLPBIO, GC11200-1) was diluted in physiological saline and used at working concentrations of 5 and 25 µmol/L. The Ca chelator EGTA (GLPBIO, GC13292-1000) was prepared in PBS with pH adjusted to 7.0–7.4 using NaOH and used at working concentrations of 5 and 20 mM. The IP3 receptor (IP3R) inhibitor 2-APB (GLPBIO, GC17562-10 mM) was diluted in physiological saline and used at working concentrations of 10 and 50 µmol/L. The mitochondrial Ca uniporter (MUC) inhibitor RU360 (SDN, R10358, 100 µg), prepared in physiological saline containing 0.5% Tween-80, was used at working concentrations of 0.025 and 0.05 µg/µL. Piezo channel-modulating drugs: The Piezo channel inhibitor GsMTx4 (GLPBIO, GC38465-500) was prepared with sterile saline and administered at doses of 1 µg (low dose) and 5 µg (high dose) per injection, with an equal volume of saline used as the solvent control. The Piezo1-specific agonist Yoda1 (GLPBIO, GC18233-5) was prepared with 1% DMSO saline and administered at doses of 0.5 µg and 2.5 µg per injection. ADO A1 receptor-modulating drugs: The A1 receptor antagonist DPCPX (GLPBIO, GC16099-25 mg) was prepared with normal saline and used at working concentrations of 0.1 and 0.5 µg/µL. The A1 receptor agonist CPA (N⁶-cyclopentyladenosine, GLPBIO, GC14369-5 mg) was prepared with normal saline and administered at a working concentration of 0.5 µg/µL. All drugs were freshly prepared before use, with the final concentration of DMSO-containing solvent not exceeding 1%. A 20-µL microsyringe was used for ST36 acupoint injection, with a total injection volume of 20 µL each time. The injection was slowly administered at two points. After administering the injection, the needle hole was gently pressed to prevent drug leakage.

### Electromyography

Electromyographic activity of the tibialis anterior muscle in the mice was recorded using needle electrodes. Before the experiment, the anesthetized mice were placed in a supine position, with the hair of the bilateral lower limbs removed. A pair of needle electrodes was inserted parallelly into the muscle belly of the tibialis anterior below the ST36 acupoint area, while the reference electrode was placed on the mouse’s tail. The electrodes were connected to a Power Lab 8/35 multichannel physiological signal acquisition system, and raw electromyographic signals were continuously recorded using LabChart 8.0 software, with key time points (such as acupuncture stimulation) marked. Following digital bandpass filtering (10–500 Hz) of the raw signals, the average integrated electromyography (iEMG) values were calculated for each experimental phase.

### Transmission electron microscopy

To observe the ultrastructure of mitochondria and SR in the muscle cells of the acupoint region, the tissue samples previously fixed with 4% glutaraldehyde were prepared for TEM. After the samples were rinsed three times (15 min each) with 0.1 M phosphate buffer (pH 7.4), they were post-fixed with 1% osmium tetroxide (Ted Pella Inc, 18456) for 2 h at room temperature in the dark, followed by three additional rinses with the same buffer. Subsequently, the samples were dehydrated using a graded ethanol series, followed by two transfers (15 min each) in 100% acetone (Sinopharm, 10000418). After dehydration, the samples were infiltrated and embedded with gradient epoxy resin 812 (SPI, 90529-77-4). Semi-thin sections of 1.5 µm were first prepared and stained with toluidine blue for orientation. Subsequently, ultrathin sections of 60–80 nm were prepared and stained with 2% uranyl acetate-ethanol solution (Sinopharm, 02624-AB) for 8 min in the dark. Then, the sections were stained with 2.6% lead citrate (Sigma, 203580) for 8 min. Following double staining, the sections were dried at room temperature. Finally, the ultrastructural images of SR and mitochondria were observed and captured under a transmission electron microscope.

For quantitative analysis, SR–mitochondrial coupling was quantified on the basis of contact length and coupled perimeter ratio; mitochondrial swelling, cristae damage and vacuolation were assessed using unified criteria. Five random non-overlapping fields were acquired per sample. All image quantification was completed by two investigators blinded to group-related information.

### Enzyme-linked immunosorbent assay

The contents of ATP and ADO in the ST36 acupoint site were measured using ELISA. Approximately 50 mg of tissue was collected, and 10% tissue homogenate was prepared by adding pre-cooled saline. The homogenate was centrifuged at 3000 r/min for 10 min at 4 °C, and the supernatant was collected as the test sample. A commercial kit (mlBio, YJ925893) was used for ATP detection. A series of ATP standard solutions and 0, 400, 800, 1600, 3200, and 6400 nmol/L fivefold diluted test samples were added to the microplate wells sequentially, followed by the addition of HRP-labeled detection antibody. The plate was incubated at 37 °C for 60 min. After the samples were washed, the TMB substrate was added for 15 min for color development. The reaction was terminated, and the absorbance was measured at a wavelength of 450 nm. The sample concentration was measured on the basis of the standard curve. The ADO detection procedure is the same and was performed using the corresponding detection kit (mlBio, YJ963520) and ADO standard concentrations of 0, 2, 4, 8, 16, and 32 nmol/L. All tissue samples were prepared in duplicate, and the results are expressed as actual concentrations.

### Mitochondrial extraction

Mitochondria were isolated from fresh ST36 acupoint tissue through differential centrifugation. All procedures were performed on ice or at 4 °C. Approximately 0.1 g of tissue was rinsed with ice-cold PBS, minced, and transferred to a pre-cooled glass homogenizer. One milliliter of mitochondrial extraction reagent (E-BC-E001) was added, and the tissue was homogenized on ice. The homogenate was centrifuged at 500 × *g* for 5 min to remove cellular debris. The resulting supernatant was then centrifuged at 15,000 × *g* for 15 min to form pellets of the crude mitochondrial fraction. The pellet was resuspended in 200 μL of mitochondrial storage buffer and purified through centrifugation at 11,000 × *g* for 10 min. The final mitochondrial pellet was resuspended in 50–100 μL of storage buffer and used immediately for subsequent assays or stored at − 80 °C.

### *Two-photon microscopy *in vivo* imaging and Ca signal data analysis*

In vivo Ca imaging of GCaMP6m-transfected muscle cells at the ST36 acupoint site in the mice was performed using a Nikon AIR MP two-photon microscope system. After the mice were anesthetized, the muscle layer of the acupoint region was exposed and fixed on a custom-made holder and covered with a coverslip. Images were acquired using a 25 × water-immersion objective (NA 1.10) with 910 nm excitation light at a resolution of 512 × 512 pixels and a rate of 30 frames/s. Ca signal dynamics were recorded for 1 min before EA stimulation, 2 min during stimulation, and 2 min after stimulation. The original. nd2 data were converted to TIFF format using ImageJ, motion artifacts were corrected using the MATLAB Mosaic toolkit, and single-cell fluorescence intensities were extracted using the Fluoro SNNAP analysis package. Ca transients were calculated using the formula ΔF/F0 = (F-F0)/F0. The firing frequency during each stimulation phase was statistically analyzed. The rise/decay time was measured, and the intercellular signal synchronization coefficient was computed.

### Statistical analysis

Data are expressed as the mean ± standard error of the mean (SEM). Statistical analysis was performed using SPSS version 19.0 software. For single-time-point measured data with normal distribution and homogeneous variance, one-way analysis of variance (ANOVA) was employed, followed by the LSD post hoc test for multiple comparisons. Dunnett’s T3 test was applied to datasets with heterogeneous variance. Non-normally distributed data were analyzed using corresponding non-parametric tests. For repeatedly measured behavioral data collected at multiple time points, repeated-measures ANOVA was used to match the nested repeated sampling characteristics, consistent with the requirement for hierarchical statistical analysis. P < 0.05 was considered to indicate statistical significance, and P < 0.01 was deemed highly significant. All behavioral pain testing and data statistical analysis were performed by independent researchers blinded to group allocation. The experimenters who collected data had no access to the grouping information of each mouse during the whole experimental process.

## Results

### Myocyte-specific Piezo1 knockdown abrogates acupuncture-induced analgesia and CaM/CaMKII signaling activation

**Fig. 1:**
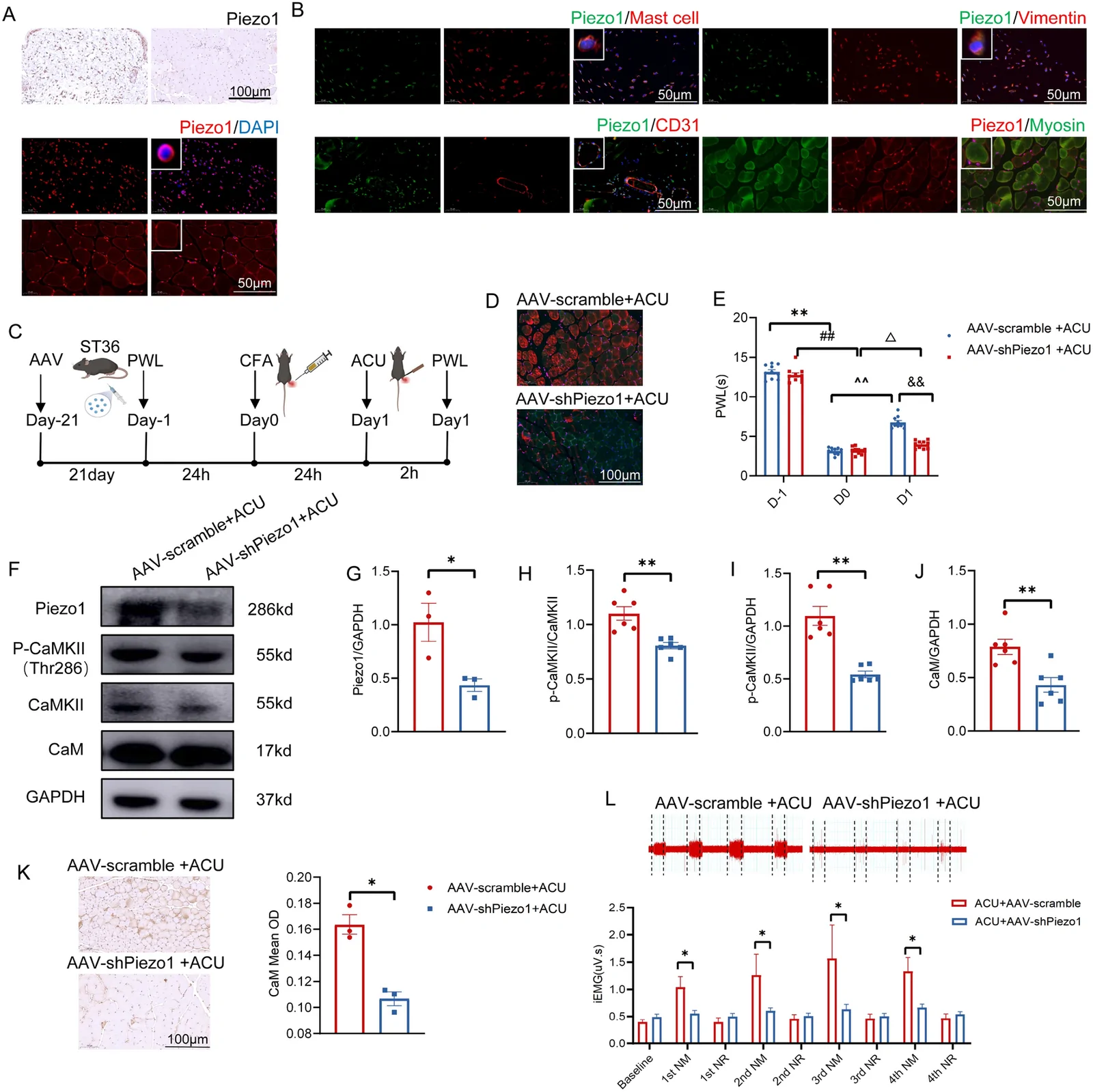
Piezo1 mediates acupuncture analgesia and regulates the CaM/CaMKII pathway. **A** IHC and IF staining revealed Piezo1 expression in the non-muscle and muscle layers of the ST36 acupoint region (scale bars = 100 µm and 50 µm, respectively). **B** Cellular co-localization analysis of Piezo1 expression. Piezo1 (green/red) co-localizes with tryptase, vimentin, and CD31 (red) in the non-muscle layer and with myosin (green) in the muscle layer, DAPI (blue). Scale bar = 50 µm. **C** Schematic illustration of the experimental timeline for viral intervention. **D** IF validation of myocyte-specific Piezo1 knockdown. DAPI (blue) and Piezo1 (green). Scale bar = 100 µm. **E** Changes in PWL before and after acupuncture in the indicated groups. Data are presented as the mean ± SEM, as analyzed by unpaired Student’s *t*-test (**P < 0.01 vs. AAV-scramble + ACU baseline; ^##^P < 0.01 vs. AAV-shPiezo1 + ACU baseline; ^^^^P < 0.01 vs. AAV-scramble + ACU post-modeling; ^△^P < 0.05 vs. AAV-shPiezo1 + ACU post-modeling; ^&&^P < 0.01 vs. AAV-scramble + ACU post-acupuncture; n = 6). **F**–**J** WB analysis of Piezo1/GAPDH, p-CaMKII/CaMKII, p-CaMKII/GAPDH, and CaM/GAPDH ratios in the muscle layer. Data are presented as the mean ± SEM, as analyzed by unpaired Student’s *t*-test (^*^P < 0.05, ^**^P < 0.01 vs. AAV-scramble + ACU; n = 6). **K** IHC validation and quantification of CaM expression in the muscle layer. Data are presented as the mean ± SEM, as analyzed by unpaired Student’s *t*-test (^*^P < 0.05 vs. AAV-scramble + ACU; scale bar = 200 µm; n = 3). **L** Comparison of iEMG values during acupuncture. Data are presented as the mean ± SEM, as analyzed by unpaired Student’s *t*-test (^*^P < 0.05 vs. AAV-scramble + ACU; n = 4)

We hypothesized that acupuncture-generated mechanical stimuli activate the mechanosensitive channel Piezo1 on myocytes at the ST36 acupoint, triggering extracellular Ca^2^⁺ influx to initiate the downstream CaM/CaMKII signaling cascade and produce analgesic effects. The expression and cellular localization of Piezo1 at the ST36 acupoint were first characterized. IHC and IF staining revealed that Piezo1 protein was expressed in both the non-muscle layer (e.g., connective tissue, vascular interstitium) and the muscle layer (muscle fiber bundles) of the acupoint region. The signal, appearing as brownish-yellow granules, was localized to the cell membrane and cytoplasm, suggesting a functional distribution consistent with a role in mechanosensation (Fig. 1A). IF double-labeling further confirmed that Piezo1 colocalized with mast cells (tryptase⁺), fibroblasts (vimentin⁺), and vascular endothelial cells (CD31⁺) in the non-muscle layer, whereas it was highly colocalized with the myocyte marker myosin in the muscle layer (Fig. 1B). Notably, extensive Piezo1 expression across multiple somatic cell types suggests that non-myocyte Piezo1 potentially participates in the modulation of acupoint signals, which could not be fully ruled out by the current myocyte-specific knockdown design. This expression profile provided a histological basis for subsequent myocyte-targeting functional studies. To specifically validate the role of myocyte Piezo1 in acupuncture-induced analgesia, myocyte-specific AAV-shRNA was used for Piezo1 expression knockdown at the ST36 acupoint (experimental timeline in Fig. 1C). IF validation confirmed a marked reduction in the Piezo1 signal (red fluorescence) in the muscle layer of the knockdown group (AAV-shPiezo1 + ACU) compared with the scramble control group (AAV-scramble + ACU) (Fig. 1D). Behaviorally, Piezo1 knockdown alone caused no alterations in baseline thermal pain thresholds under inflammatory conditions. Acupuncture significantly restored the PWL in the control group, but this analgesic effect was markedly attenuated (by 41.1%) in the Piezo1 knockdown group (Fig. 1E). WB confirmed efficient Piezo1 knockdown (57.5% reduction in the Piezo1/GAPDH ratio, Fig. 1F–J) and, importantly, revealed that this knockdown significantly inhibited acupuncture-induced CaMKII phosphorylation and CaM expression upregulation in the muscle layer (Fig. 1F–J). Consistent with WB results, IHC showed that CaM protein levels in the muscle layer were reduced in the knockdown group (Fig. 1K). Furthermore, electromyographic recording demonstrated that needling-elicited iEMG activity was significantly lower in the Piezo1 knockdown group than in the control group (Fig. 1L), indicating the regulatory role of Piezo1 in acupuncture-induced local muscle electrical activity. Thus, Piezo1 is expressed in myocytes and other cell types at ST36. Specific Piezo1 knockdown in myocytes significantly inhibits the acupuncture-induced analgesic effect, activation of the downstream CaM/CaMKII pathway, and the local electromyographic response. These findings indicate that myocyte Piezo1 serves as a critical upstream contributor that transforms acupuncture-induced mechanical stimulation into intracellular biological signals to trigger the subsequent CaM/CaMKII cascade.

### Pharmacological manipulation of Piezo1 regulates acupuncture-induced analgesia and CaM/CaMKII pathway activity

**Fig. 2:**
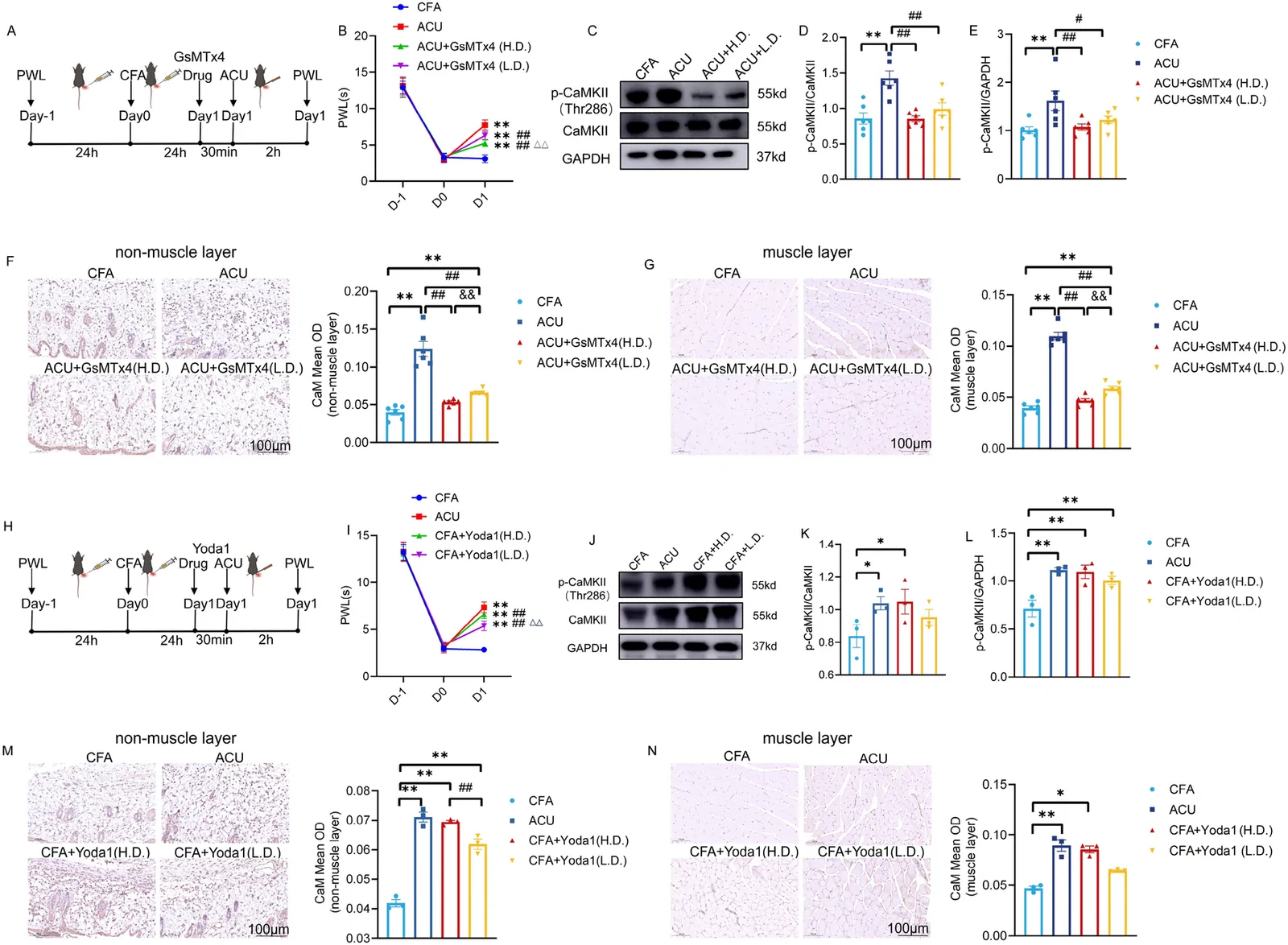
Pharmacological validation of Piezo1 channel function. **A** Schematic illustration of the experimental timeline for GsMTx4 intervention. **B** Antagonistic effect of GsMTx4 on acupuncture analgesia. Data are presented as the mean ± SEM, as analyzed by one-way ANOVA followed by post-hoc test (^**^P < 0.01 vs. CFA; ^##^P < 0.01 vs. ACU; ^△△^P < 0.01 vs. ACU + GsMTx4 (H.D.); n = 9). **C**–**E** Effect of GsMTx4 on CaMKII phosphorylation in acupoint tissues. Data are presented as the mean ± SEM, as analyzed by one-way ANOVA followed by post-hoc test (^**^P < 0.01 vs. CFA; ^#^P < 0.05, ^##^P < 0.01 vs. ACU; n = 6). **F**, **G** IHC staining and analysis demonstrated the effect of GsMTx4 on CaM protein expression in the non-muscle (**F**) and muscle (**G**) layers. Data are presented as the mean ± SEM, as analyzed by one-way ANOVA followed by post-hoc test (^**^P < 0.01 vs. CFA; ^##^P < 0.01 vs. ACU; ^&&^P < 0.01 vs. ACU + GsMTx4 (H.D.); n = 6). **H** Schematic representation of the experimental timeline for Yoda1 intervention. **I** Analgesic effect of Yoda1 mimicking acupuncture. Data are presented as the mean ± SEM, as analyzed by one-way ANOVA followed by post-hoc test (^**^P < 0.01 vs. CFA; ^##^P < 0.01 vs. ACU; ^△△^P < 0.01 vs. CFA + Yoda1 (H.D.); n = 9). **J**–**L** The effect of Yoda1 on CaMKII phosphorylation in acupoint tissues. Data are presented as the mean ± SEM, as analyzed by one-way ANOVA followed by post-hoc test (^*^P < 0.05, ^**^P < 0.01 vs. CFA; n = 3). **M**, **N** The effect of Yoda1 on CaM protein expression in the non-muscle (**M**) and muscle (**N**) layers. Data are presented as the mean ± SEM, as analyzed by a non-parametric test (^*^P < 0.05, ^**^P < 0.01 vs. CFA; ^##^P < 0.01 vs. ACU; n = 3)

To further verify the function of Piezo1, bidirectional pharmacological interventions were administered with the mechanosensitive channel inhibitor GsMTx4 and Piezo1 agonist Yoda1. Local pretreatment with GsMTx4 (Fig. 2A) concentration-dependently attenuated the acupuncture-induced analgesic effect (Fig. 2B). Concurrently, it inhibited the acupuncture-induced increase in CaMKII phosphorylation (Fig. 2C–E) and upregulation of CaM expression in the non-muscle and muscle layers of the acupoint (Fig. 2F, G). Thus, blocking Piezo1 activity impairs acupuncture-triggered CaM/CaMKII signaling. Conversely, local administration of Yoda1 (Fig. 2H) partially mimicked the acupuncture-induced analgesic effect in a concentration-dependent manner (Fig. 2I). Yoda1 also elevated CaMKII phosphorylation (Fig. 2J–L) and increased CaM expression (Fig. 2M, N), recapitulating acupuncture-triggered key molecular events. Taken together, these gain- and loss-of-function pharmacological results reveal that Piezo1 participates in the modulation of the CaM/CaMKII pathway and contributes to acupuncture-induced analgesia at ST36.

### ***CaM acts as a downstream mediator of Piezo1-mediated Ca***^***2***^***⁺ influx to drive CaM/CaMKII activation and acupuncture-induced analgesia at ST36***

To further clarify the mechanistic association between Piezo1-mediated signaling and CaM/CaMKII activation, we focused on CaM as a key Ca sensor that transmits Ca^2^⁺ signals to trigger CaMKII phosphorylation. The central hypothesis that CaM serves as a critical downstream mediator through which Piezo1 promotes acupuncture-induced analgesia and activates the CaM/CaMKII signaling pathway was tested. First, the acupuncture-induced analgesic effect at the ST36 acupoint was evaluated in a CFA-induced arthritis mouse model (experimental timeline in Supplement Fig. S3A). PWL measurements revealed that deep, but not shallow, needling at ST36 significantly reversed CFA-induced inflammatory hyperalgesia (Supplement Fig. S3B), indicating a depth-dependent analgesic effect. Conversely, acupuncture treatment at ST25 caused no significant alterations in PWL values (Supplement Fig. S3C), suggesting that the analgesic effect is specific to deep needling at ST36. We further analyzed CaM/CaMKII pathway activation in different tissue layers of ST36. In the non-muscle layer, deep and shallow needling both increased CaMKII phosphorylation (Supplement Fig. S3D–F). By contrast, within the muscle layer, CaMKII phosphorylation was significantly upregulated only by deep acupuncture (Supplement Fig. S3G–I), suggesting a tissue-specific activation of this key signaling node. Next, the expression of CaM, the upstream Ca sensor, was investigated. IHC revealed that both needling depths elevated CaM levels in the non-muscle layer, but only deep acupuncture robustly increased CaM expression within the muscle layer (Supplement Fig. S3J, K). These results indicate that deep acupuncture at ST36 effectively alleviates inflammatory hyperalgesia and is uniquely associated with the upregulation of both CaM expression and CaMKII phosphorylation specifically within the muscle layer of the acupoint.

**Fig. 3:**
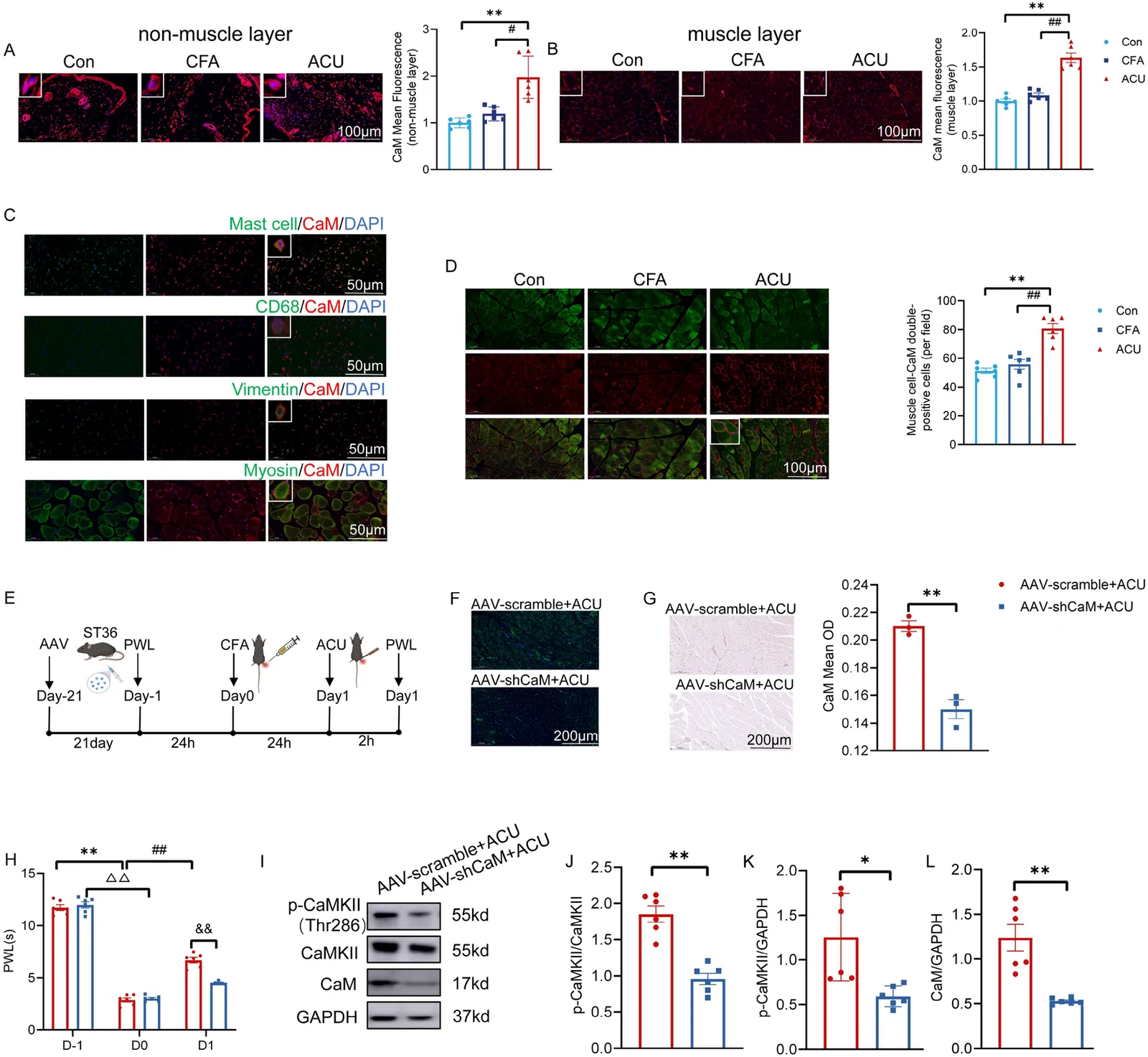
Validation of CaM as a downstream mediator of Piezo1 in acupuncture analgesia. **A**, **B** Representative IF images displaying CaM expression (red) and DAPI (blue, nuclear staining) in the non-muscle and muscle layers of the ST36 acupoint region, and the quantification of the mean fluorescence intensity of CaM in these two layers. Data are presented as the mean ± SEM, as analyzed by one-way ANOVA followed by post-hoc test (^**^P < 0.01 vs. Con group; ^#^P < 0.05, ^##^P < 0.01 vs.CFA group; n = 6). **C** Co-localization analysis of CaM (red) with cell-specific markers (green: Tryptase, CD68, Vimentin, Myosin) and DAPI (blue). Scale bar = 100 µm. **D** Quantification of CaM/Myosin double-positive cells in the muscle layer. Data are presented as the mean ± SEM, as analyzed by one-way ANOVA followed by post-hoc test (^*^P < 0.01 vs. Con group; ^##^P < 0.01 vs.CFA group; n = 6; scale bar = 100 µm). **E** Schematic illustration of the experimental timelines for viral and pharmacological interventions (over a 21-day intervention period), followed by assessments, Day -1 (baseline before acupuncture), Day 0 (CFA immunization for arthritis induction), and Day 1 (acupuncture intervention). **F** IF validation of CaM knockdown in myocytes at the acupoint (DAPI, blue; CaM, green; scale bar = 200 μm). **G** IHC validation and quantification of CaM knockdown. Data are presented as the mean ± SEM, as analyzed by unpaired Student’s *t*-test (^**^P < 0.01 vs. AAV-scramble + ACU; scale bar = 200 µm; n = 3). **H** Changes in PWL before and after acupuncture in the indicated groups. Data are presented as the mean ± SEM, as analyzed by unpaired Student’s *t*-test (^**^P < 0.01 vs. AAV-scramble + ACU baseline; ^△△^P < 0.01 vs. AAV-shCaM + ACU baseline; ^##^P < 0.01 vs. AAV-scramble + ACU post-modeling; ^&^P < 0.01 vs. AAV-scramble + ACU post-acupuncture; n = 6). **I**–**L** WB analysis of p-CaMKII/CaMKII, p-CaMKII/GAPDH, and CaM/GAPDH ratios in the muscle layer. Data are presented as the mean ± SEM, as analyzed by unpaired Student’s *t*-test (^*^P < 0.05, ^**^P < 0.01 vs. AAV-scramble + ACU; n = 6)

IF quantification of the CaM signal intensity further confirmed this muscle-specific increase in CaM levels (Fig. 3A, B). IF colocalization studies further defined cellular CaM distribution at ST36 (Fig. 3C). CaM was present in non-muscle cell types (e.g., mast cells, macrophages, and fibroblasts) and exhibited pronounced, fiber-aligned expression within myocytes. Quantitative analysis confirmed that deep acupuncture specifically increased the number of CaM-positive myocytes in the muscle layer (Fig. 3D). To genetically verify the function of CaM in acupuncture-induced analgesia and CaMKII activation, we used AAV-shRNA and knocked down myocyte-specific CaM at ST36 (Fig. 3E). IF and IHC quantification confirmed that CaM expression was significantly reduced in the muscle layer of the CaM knockdown group (AAV-shCaM + ACU) compared with the scramble control group (AAV-scramble + ACU) (Fig. 3F, G). Behavioral tests revealed that, while acupuncture significantly restored the PWL in the scramble control group, this analgesic effect was markedly attenuated in the CaM knockdown group (Fig. 3H). According to WB analysis, CaM knockdown significantly inhibited acupuncture-induced CaMKII phosphorylation in the muscle layer (Fig. 3I–L). Collectively, these findings demonstrate that CaM functions as a critical downstream mediator of Piezo1-initiated Ca^2^⁺ signaling, which is required for muscle-specific CaM/CaMKII activation and subsequent acupuncture-induced analgesia at ST36.

### ***Intracellular Ca***^***2***^***⁺ acts as the key upstream signal to activate the CaM/CaMKII pathway and mediate acupuncture analgesia***

**Fig. 4:**
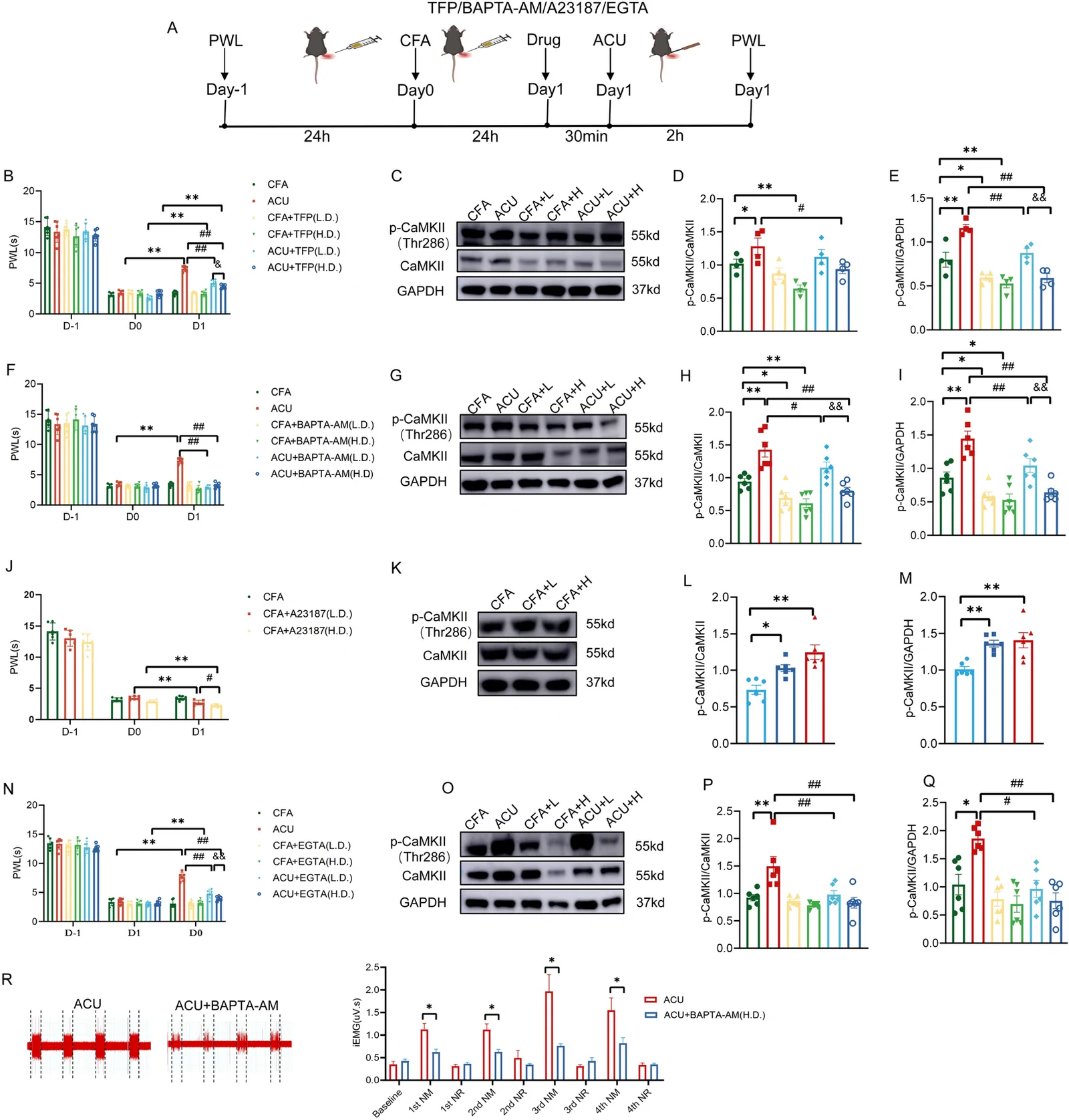
Intracellular Ca^2^⁺ regulates acupuncture analgesia and the CaM/CaMKII pathway. **A** Timeline for pharmacological intervention experiments. **B** The antagonistic effect of TFP on acupuncture analgesia (^**^P < 0.01 vs. CFA; ^##^P < 0.01 vs. ACU; ^&^P < 0.05 vs. ACU + TFP (H.D.); n = 6). **C**–**E** Effect of TFP on CaMKII phosphorylation in the muscle layer. Data are presented as the mean ± SEM, as analyzed by one-way ANOVA followed by post-hoc test (^*^P < 0.05, ^**^P < 0.01 vs. CFA; ^#^P < 0.05, ^##^P < 0.01 vs. ACU; ^&&^P < 0.01 vs. ACU + TFP (H.D.); n = 4). **F** The antagonistic effect of BAPTA-AM on acupuncture analgesia (^**^P < 0.01 vs. CFA; ^##^P < 0.01 vs. ACU; n = 6). **G**–**I** Effect of BAPTA-AM on CaMKII phosphorylation. Data are presented as the mean ± SEM, as analyzed by one-way ANOVA followed by post-hoc test (^*^P < 0.05, ^**^P < 0.01 vs. CFA; ^#^P < 0.05, ^##^P < 0.01 vs. ACU; ^&&^P < 0.01 vs. ACU + BAPTA-AM (H.D.); n = 6). **J** Effect of A23187 on the basal pain threshold (^**^P < 0.01 vs. CFA; ^#^P < 0.05 vs. CFA + A23187 (H.D.); n = 6). **K**–**M** Effect of A23187 on CaMKII phosphorylation. Data are presented as the mean ± SEM, as analyzed by one-way ANOVA followed by post-hoc test (^*^P < 0.05, ^**^P < 0.01 vs. CFA; n = 6). **N** The antagonistic effect of EGTA on acupuncture analgesia (^**^P < 0.01 vs. CFA; ^##^P < 0.01 vs. ACU; ^&&^P < 0.01 vs.ACU + EGTA (H.D.); n = 6). **O**–**Q** Effect of EGTA on CaMKII phosphorylation in the muscle layer. Data are presented as the mean ± SEM, as analyzed by one-way ANOVA followed by post-hoc test (^*^P < 0.05, ^**^P < 0.01 vs. CFA; ^#^P < 0.05, ^##^P < 0.01 vs. ACU; n = 6). **R** Effect of BAPTA-AM on acupuncture-induced EMG activity. Data are presented as the mean ± SEM, as analyzed by unpaired Student’s *t*-test (P < 0.05 vs. ACU; n = 4). ACU: deep acupuncture; L.D.: low dose; H.D.: high dose

To further verify the aforementioned results, we locally pretreated the ST36 acupoint with TFP, a CaM antagonist (Fig. 4A). TFP pretreatment concentration-dependently attenuated acupuncture-induced analgesia (Fig. 4B) and reduced the acupuncture-induced upregulation of CaMKII phosphorylation (Fig. 4C–E). These pharmacological results are consistent with the data obtained from CaM genetic knockdown, further supporting the essential role of the CaM-CaMKII-signaling axis. Next, the upstream regulator of CaM was investigated by bidirectionally manipulating intracellular Ca^2^⁺ levels. Intracellular Ca^2^⁺ chelation with BAPTA-AM markedly reduced the acupuncture-induced analgesic effects and subsequent CaMKII activation (Figs. 4F–I). Conversely, increasing intracellular Ca^2^⁺ levels with the Ca^2^⁺ ionophore A23187 exacerbated inflammatory hyperalgesia and directly elevated CaMKII phosphorylation (Fig. 4J–M). This complementary gain- and loss-of-function evidence establishes intracellular Ca^2^⁺ as the key upstream trigger that activates the CaM-CaMKII pathway following acupuncture. Furthermore, bidirectional manipulation of intracellular Ca^2^⁺ levels correspondingly modulates pain behavior and CaMKII phosphorylation. These results collectively indicate that acupuncture exerts analgesic effects at ST36 by elevating intracellular Ca^2^⁺ levels, thereby activating the CaM/CaMKII-signaling axis. To ascertain that the Ca^2^⁺ signal originates from the extracellular space, EGTA was used to chelate extracellular Ca^2^⁺. EGTA pretreatment concentration-dependently antagonized the acupuncture-induced analgesic effect (Fig. 4N) and suppressed the associated increase in CaMKII phosphorylation (Fig. 4O–Q). This finding demonstrates that extracellular Ca^2^⁺ influx is critical for initiating the signal. Finally, electromyographic recording indicated that intracellular Ca^2^⁺ chelation also reduced acupuncture-induced increases in iEMG activity at the acupoint (Fig. 4R), suggesting that Ca^2^⁺ signaling contributes to the local electrophysiological responses of muscle tissue elicited by needling. In summary, our results reveal that acupuncture intervention at ST36 increases intracellular Ca^2^⁺ levels through extracellular Ca^2^⁺ influx, thereby activating the CaM/CaMKII-signaling axis and ultimately producing analgesic effects.

### *Acupuncture enhances SR–mitochondrial Ca signaling and energy metabolism *via* the IP3R-GRP75-VDAC1-MCU pathway*

**Fig. 5:**
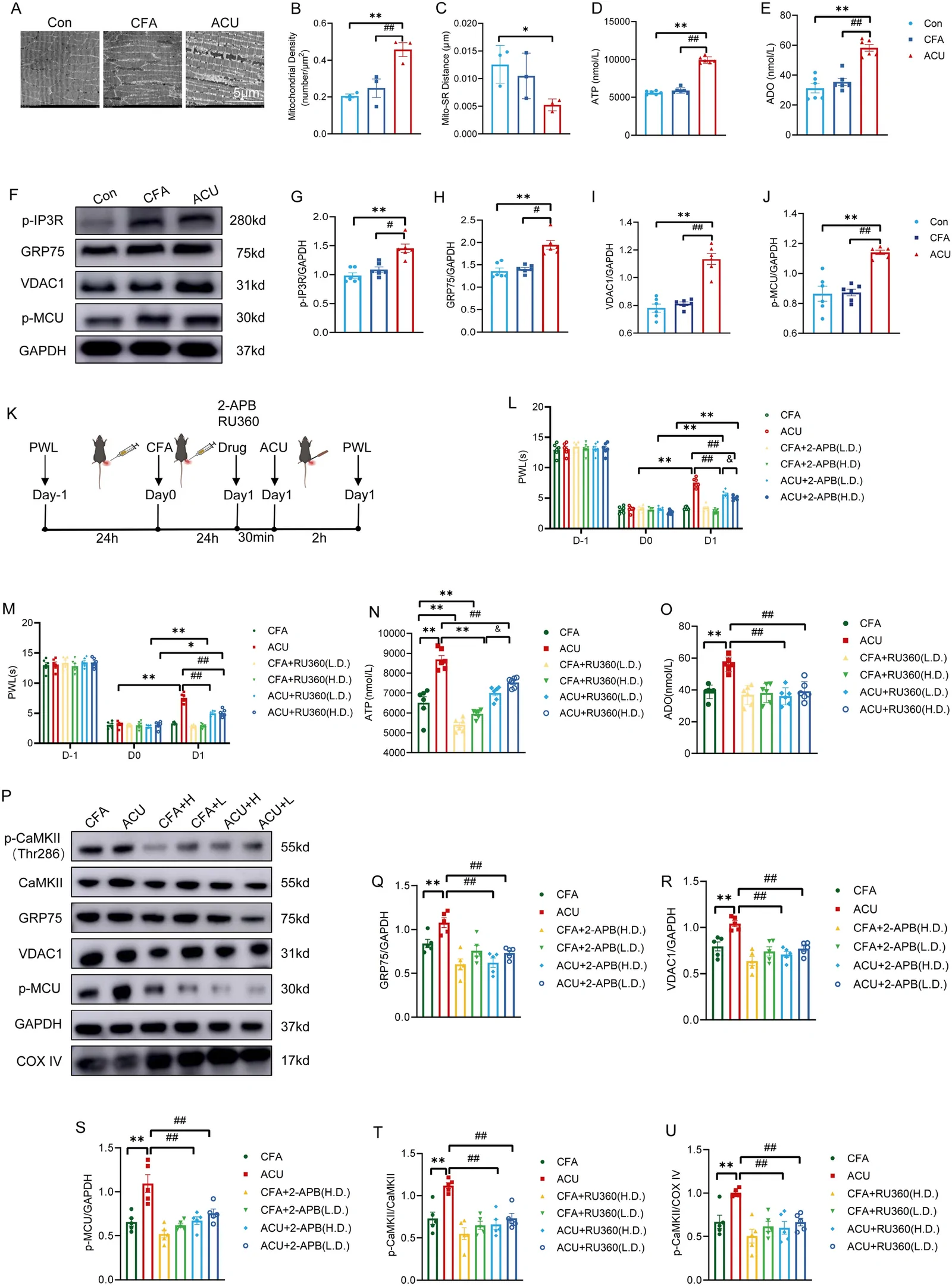
Acupuncture modulates SR-mitochondrial function via the IP3R-GRP75-VDAC1-MCU pathway. **A**–**C** Transmission electron microscopy images of myocytes in the acupoint region after acupuncture. Data are presented as the mean ± SEM, as analyzed by one-way ANOVA followed by post-hoc test (^*^P < 0.05, ^**^P < 0.01 vs. Con; ^##^P < 0.01 vs. CFA; scale bar = 5 µm; n = 3). **D**, **E** ATP (**D**) and ADO (**E**) content in acupoint tissues. Data are presented as the mean ± SEM, as analyzed by one-way ANOVA followed by post-hoc test (^**^P < 0.01 vs. Con; ^##^P < 0.01 vs. CFA; n = 6). **F**–**J** WB analysis of p-IP3R/GAPDH, GRP75/GAPDH, VDAC1/GAPDH, and p-MCU/GAPDH ratios in acupoint muscle tissues. p-IP3R and GRP75 were analyzed via Kruskal–Wallis H-test with Dunn’s multiple comparisons; VDAC1 and p-MCU were analyzed by one-way ANOVA followed by post-hoc test (^**^P < 0.01 vs. Con; ^#^P < 0.05, ^##^P < 0.01 vs. CFA; n = 3–6). **K** Schematic illustration of the experimental timeline for pharmacological interventions. **L** Effect of the IP3R inhibitor 2-APB on acupuncture analgesia. Data are presented as the mean ± SEM, as analyzed by one-way ANOVA followed by post-hoc test (^**^P < 0.01 vs. CFA; ^##^P < 0.01 vs. ACU; ^&^P < 0.05 vs. ACU + 2-APB (H.D.); n = 6). **M** Effect of the MCU inhibitor Ru360 on acupuncture analgesia. Data are presented as the mean ± SEM, as analyzed by one-way ANOVA followed by post-hoc test (^*^P < 0.05, ^**^P < 0.01 vs. CFA; ^##^P < 0.01 vs. ACU; n = 6). **N**, **O** ATP (**N**) and ADO (**O**) content in acupoint tissues. Data are presented as the mean ± SEM, as analyzed by one-way ANOVA followed by post-hoc test. **O** Content in acupoint tissues (^*^P < 0.05, ^**^P < 0.01 vs. CFA; ^##^P < 0.01 vs. ACU; ^&^P < 0.05 vs. ACU + Ru360 (H.D.); n = 6). **P**–**S** Effect of 2-APB on the protein expression of GRP75, VDAC1, and p-MCU. Data are presented as the mean ± SEM, as analyzed by one-way ANOVA followed by post-hoc test (^**^P < 0.01 vs. CFA; ^##^P < 0.01 vs. ACU; n = 5). **T**, **U** Effect of Ru360 on the ratios of p-CaMKII/CaMKII (**T**) and p-CaMKII/COX IV (**U**) in mitochondria. Data are presented as the mean ± SEM, as analyzed by one-way ANOVA followed by post-hoc test (^**^P < 0.01 vs. CFA; ^##^P < 0.01 vs. ACU; n = 5)

Based on the understanding of the established initiating role of the Piezo1/Ca^2^⁺/CaM/CaMKII axis, we investigated the downstream amplification of the Ca signal. We proposed that acupuncture triggers initial Ca^2^⁺ influx through Piezo1, thereby stimulating Ca^2^⁺ release from the SR and subsequent Ca^2^⁺ transport into mitochondria via the IP3R-GRP75-VDAC1-MCU complex. This coordinated signaling enhances mitochondrial bioenergetics and ADO production, ultimately increasing the analgesic effect. TEM revealed that acupuncture significantly increased mitochondrial density and altered the SR–mitochondria coupling distance in the muscle tissues of the ST36 acupoint region (Fig. 5A–C), suggesting increased structural and functional coupling between these organelles. Consistent with improved mitochondrial function, ELISA revealed that acupuncture increased both ATP and ADO levels at the acupoint site (Fig. 5D, E). At the molecular level, acupuncture upregulated key components of the SR–mitochondrial Ca transfer pathway, increasing p-IP3R, GRP75, VDAC1, and p-MCU levels in the muscle layer (Fig. 5F–J). To verify the functional contribution of IP3R to this signaling cascade, the IP3R inhibitor 2-APB was locally administered to the ST36 acupoint (Fig. 5K). Pharmacological IP3R blockade dose-dependently attenuated acupuncture-induced analgesia (Fig. 5L) and reduced acupuncture-mediated upregulation of GRP75, VDAC1, and p-MCU (Fig. 5P–S). These results support that IP3R-dependent Ca^2^⁺ release from the SR is required for activating the SR–mitochondrial Ca transfer axis. Similarly, inhibition of the MCU with RU360 (Fig. 5K) partially antagonized acupuncture-induced analgesia (Fig. 5M). RU360 treatment also inhibited acupuncture-induced increases in ATP and ADO levels (Fig. 5N, O) and reduced mitochondrial CaMKII activation (Fig. 5T, U), indicating that MCU-mediated mitochondrial Ca^2^⁺ uptake is crucial for the metabolic and signaling amplification of the analgesic response. In conclusion, acupuncture amplifies analgesic signaling by strengthening SR–mitochondrial Ca crosstalk via the IP3R-GRP75-VDAC1-MCU axis, which increases local ATP and ADO accumulation and activates mitochondrial CaMKII. Because the current study focuses primarily on upstream Ca metabolic regulation, CD39/CD73-mediated ATP–ADO conversion and intracellular/extracellular purinergic signaling were not characterized. Therefore, the downstream ATP–ADO-signaling axis is proposed based on altered metabolite levels and needs to be further verified in the future. Collectively, our results demonstrate that SR–mitochondrial Ca signaling is critical for acupuncture-induced metabolic amplification and analgesia.

### Piezo1 regulates the downstream SR-mitochondrial pathway and ADO-mediated analgesic output

**Fig. 6:**
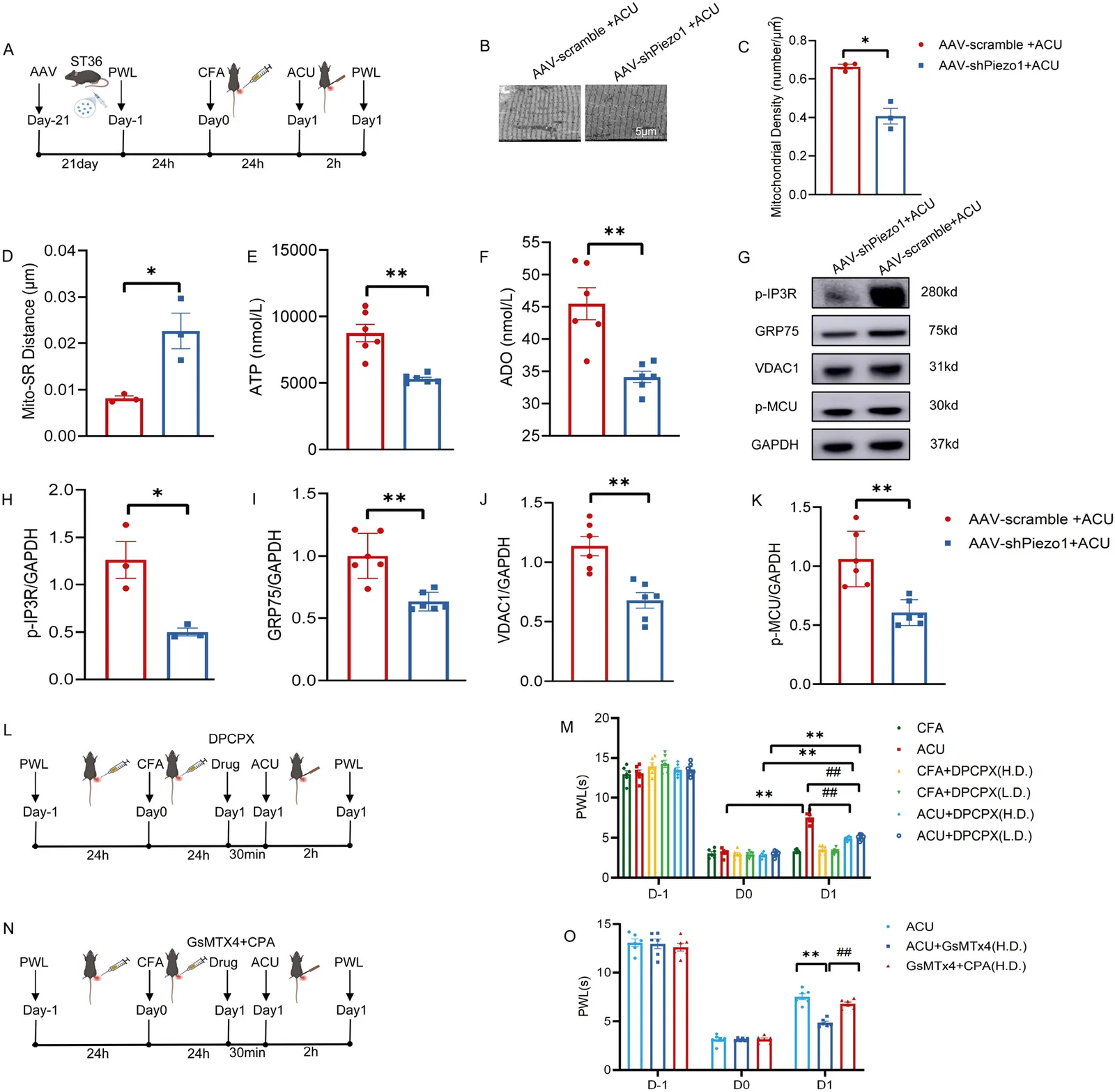
Functional validation of Piezo1 in regulating the downstream pathway and ADO-mediated analgesia. **A** Schematic illustration of the experimental timeline for Piezo1 knockdown. **B**–**D** Ultrastructure of myocytes in the acupoint region observed by transmission electron microscopy (**B**), with the quantification of mitochondrial density (**C**) and SR-mitochondria distance (**D**). Data are presented as the mean ± SEM, as analyzed by unpaired Student’s *t*-test (^*^P < 0.05 vs. AAV-scramble + ACU; scale bar = 5 µm; n = 3). **E**, **F** ATP (**E**) and ADO (**F**) content in acupoint tissues. Data are presented as the mean ± SEM, as analyzed by unpaired Student’s *t*-test (^**^P < 0.01 vs. AAV-scramble + ACU; n = 6). **G**–**K** WB analysis of p-IP3R, GRP75, VDAC1, and p-MCU protein ratios in acupoint tissues. Data are presented as the mean ± SEM, as analyzed by unpaired Student’s *t*-test (^*^P < 0.05, ^**^P < 0.01 vs. AAV-scramble + ACU; n = 3–6). **L** Schematic representation of the experimental timeline for DPCPX intervention. **M** Effect of DPCPX on acupuncture analgesia. Data are presented as the mean ± SEM, as analyzed by one-way ANOVA followed by post-hoc test (^**^P < 0.01 vs. CFA; ^##^P < 0.01 vs. ACU; n = 6). **N** Schematic illustration of the experimental timeline for combined GsMTx4 and CPA intervention. **O** Effect of combined GsMTx4 and CPA intervention on acupuncture analgesia. Data are presented as the mean ± SEM, as analyzed by one-way ANOVA followed by post-hoc test (^**^P < 0.01 vs. ACU; ^##^P < 0.01 vs. ACU + GsMTx4; n = 6)

To establish the functional association between Piezo1-mediated upstream Ca influx and the downstream SR–mitochondrial cascade, myocyte-specific Piezo1 knockdown was performed (experimental timeline in Fig. 6A). Compared with the AAV-scramble + ACU group, Piezo1 knockdown (AAV-shPiezo1 + ACU) notably decreased mitochondrial density and increased the SR–mitochondrial coupling distance in the muscle tissues of the ST36 acupoint (Fig. 6B–D). This finding indicates that Piezo1 deficiency impairs the structural coupling required for organellar propagation of Ca signals. Furthermore, Piezo1 knockdown significantly reduced the acupuncture-induced increase in ATP and ADO levels at the acupoint site (Fig. 6E, F). WB confirmed that Piezo1 knockdown suppressed the acupuncture-induced upregulation of key proteins in the SR–mitochondrial Ca transfer pathway, including p-IP3R, GRP75, VDAC1, and p-MCU (Fig. 6G–K). Collectively, these findings support that Piezo1 serves as an upstream mechanosensor required for acupuncture-initiated SR–mitochondrial Ca signal amplification and subsequent metabolic enhancement. To further explore the terminal effector mechanism underlying acupuncture-induced analgesia, the ADO A1 receptor antagonist DPCPX was locally applied at ST36 (Fig. 6L). DPCPX treatment dose-dependently reduced acupuncture-induced analgesic effects (Fig. 6M), suggesting that A1 receptor activation contributes substantially to acupuncture-mediated pain relief. Finally, we conducted a rescue experiment to directly link the Piezo1-initiated pathway to ADO signaling. While GsMTx4 alone pretreatment diminished acupuncture-induced analgesia, coadministration of the ADO A1 receptor agonist CPA (Fig. 6N) with GsMTx4 significantly restored the analgesic effect (Fig. 6O). These rescue data indicate that activated downstream A1 receptor signaling functionally compensates for impaired Piezo1 activity, verifying the hierarchical Piezo1–Ca–ADO analgesic cascade. In summary, acupuncture-induced Piezo1 activation initiates Ca^2^⁺ signaling, enhances SR–mitochondrial coupling, and increases ATP and ADO levels to drive A1 receptor-mediated analgesia. The CD39/CD73 pathway and intracellular/extracellular purinergic signals remain to be clarified. Piezo1 is a critical upstream modulator of this entire signaling cascade.

### In vivo* imaging reveals that EA rapidly activates myocyte Ca signaling *via* Piezo1 to mediate analgesia*

**Fig. 7:**
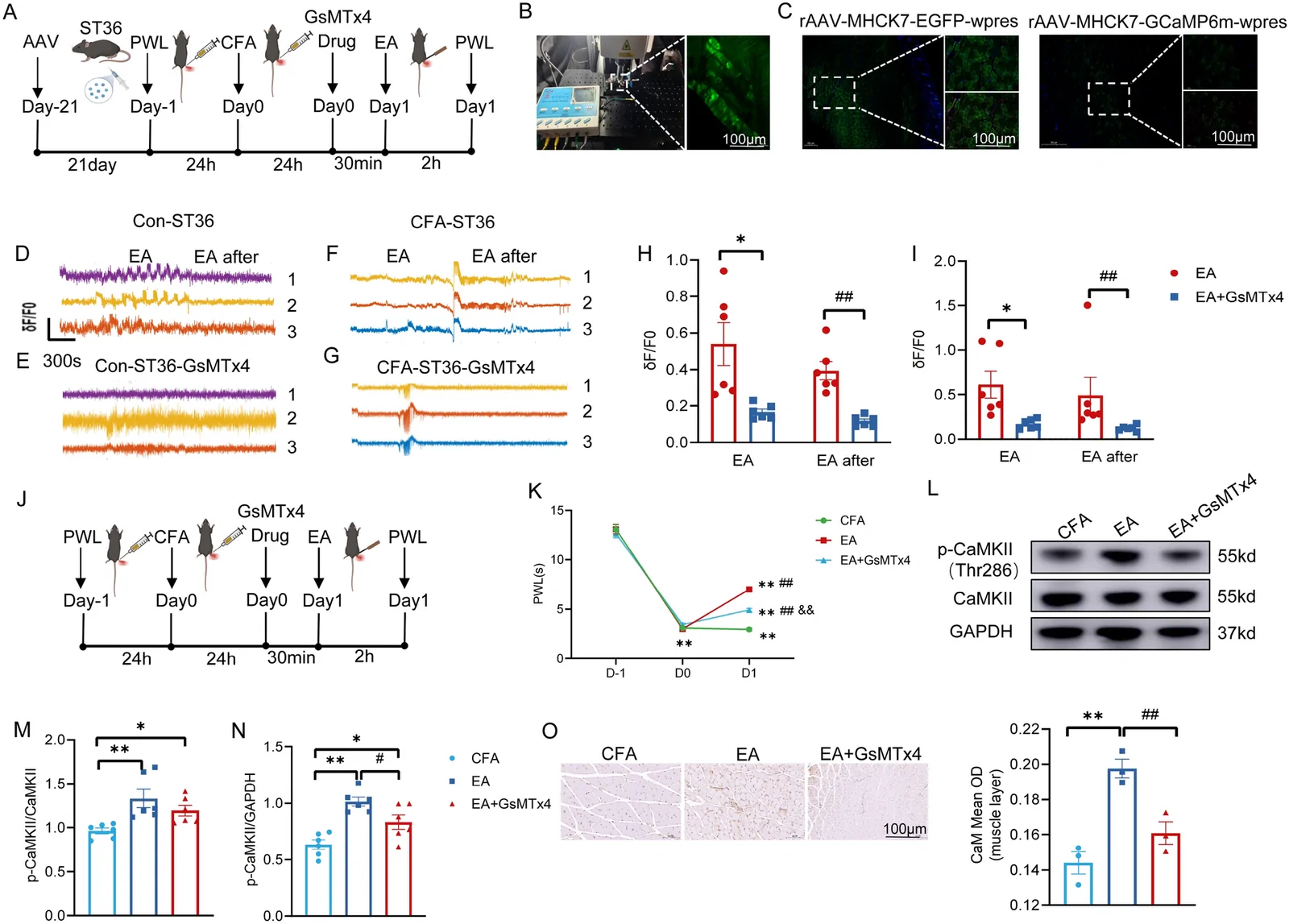
EA activates myocyte calcium (Ca) signaling and produces analgesia via Piezo1. **A** Schematic illustration of the in vivo Ca imaging experimental timeline. **B** Representative two-photon microscopy imaging field of the ST36 acupoint region. Scale bar = 100 µm. **C** IF validation of virus-specific expression in myocytes. Green: EGFP/GCaMP6m; red: myocyte marker MYH4; blue: DAPI. **D**–**G** Representative Ca signal traces (ΔF/F0) from individual myocytes during EA stimulation (gray shaded area) in: naive mice + EA (**D**), naive mice + GsMTx4 + EA (**E**), CFA mice + EA (**F**), and CFA mice + GsMTx4 + EA (**G**). **H**, **I** Quantitative comparison of ΔF/F0 values during and after EA stimulation in naive (**H**) and CFA (**I**) mice. Data are presented as the mean ± SEM, as analyzed by unpaired Student’s *t*-test (^*^P < 0.05 vs. EA group; ^##^P < 0.01 vs. after-EA period; n = 6). **J** Schematic illustration of the experimental timeline for behavioral and molecular validation with GsMTx4 pretreatment. **K** Effect of GsMTx4 on EA analgesia. Data are presented as the mean ± SEM, as analyzed by one-way ANOVA followed by post-hoc test (^**^P < 0.01 vs. baseline; ^##^P < 0.01 vs. CFA group; ^&&^P < 0.01 vs. EA group; n = 6). **L**–**N** Effect of GsMTx4 on the ratios of p-CaMKII/CaMKII (**L**, **M**) and p-CaMKII/GAPDH (**N**) in the muscle layer of the acupoint. Data are presented as the mean ± SEM, as analyzed by one-way ANOVA followed by post-hoc test (^*^P < 0.05, ^**^P < 0.01 vs. CFA group; ^#^P < 0.05 vs. EA group; n = 6). **O** Effect of GsMTx4 on CaM protein expression in the muscle layer, as assessed by IHC. Data are presented as the mean ± SEM, as analyzed by one-way ANOVA followed by post-hoc test (^**^P < 0.01 vs. CFA group; ^##^P < 0.01 vs. EA group; scale bar = 100 µm; n = 3)

**Fig. 8:**
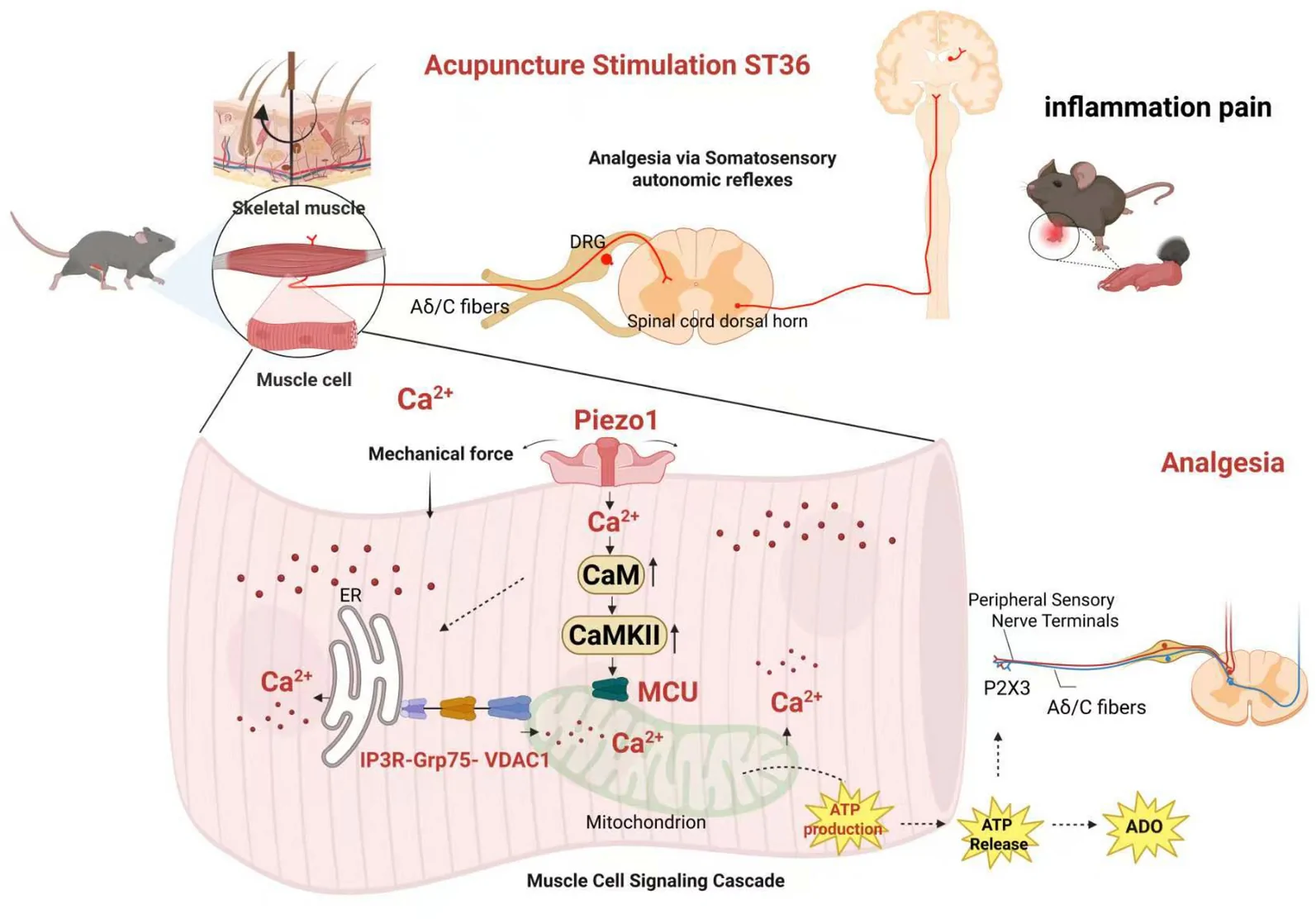
Schematic illustration of the mechanism through which acupuncture at ST36 alleviates arthritic pain via the Piezo1/Ca^2^⁺/CaM/CaMKII/ATP-signaling axis

To dynamically validate the general mechanism underlying acupuncture-induced analgesia in vivo and to characterize EA-mediated signal initiation, in vivo two-photon Ca imaging was performed to investigate real-time Ca responses in myocytes at the ST36 acupoint and the role of Piezo1 (experimental design and imaging field shown in Fig. 7A, B). First, we validated the viral tools for Ca imaging. Both the control virus (rAAV-MHCK7-EGFP) and the Ca indicator virus (rAAV-MHCK7-GCaMP6m) were specifically expressed in myocytes at the acupoint. This finding was confirmed by the high colocalization of the green fluorescence (EGFP/GCaMP6m) with the myocyte-specific marker MYH4 (red) (Fig. 7C), and established a reliable platform for dynamic Ca monitoring. Using rAAV-GCaMP6m, real-time Ca signals in myocytes were recorded during EA stimulation in both naive and CFA mice (representative traces in Fig. 7D–G). Quantitative analysis revealed that EA induced rapid, synchronized increases in cytosolic Ca (ΔF/F0) in myocytes, regardless of the inflammatory state. Notably, in both naive (Fig. 7H) and CFA mice (Fig. 7I), local pretreatment with the Piezo1 inhibitor GsMTx4 markedly diminished EA-evoked Ca transients during and after EA stimulation. According to these in vivo observations, Piezo1 contributes substantially to EA-triggered mechanosensitive Ca^2^⁺ influx in myocytes of the acupoint. We further confirmed the aforementioned findings at behavioral and molecular levels (experimental timeline in Fig. 7J). Behavioral tests unveiled that EA efficiently relieved CFA-induced inflammatory hyperalgesia, whereas GsMTx4 pretreatment partially reversed EA-mediated analgesia (Fig. 7K). At the molecular level, EA increased CaMKII phosphorylation and CaM expression in the muscle layer of the acupoint. These EA-induced changes were significantly reduced by GsMTx4 (Fig. 7L–O). In summary, in vivo Ca imaging directly demonstrates that EA rapidly activates myocyte Ca signaling in a Piezo1-dependent manner. Pharmacological blockade of Piezo1 reduces EA-triggered Ca transients, weakens EA analgesic behaviors, and inhibits downstream CaM/CaMKII pathway activation. Collectively, these findings support that Piezo1 functions as a key upstream mechanosensor that mediates EA-initiated Ca signaling and subsequent analgesic responses (Fig. 8).

## Discussion

Acupuncture-induced analgesia, an effective pain management strategy with minimal side effects, has gained widespread acceptance in clinical practice. Numerous clinical and preclinical studies have confirmed that acupuncture rapidly alleviates acute inflammatory pain. However, the peripheral initiation mechanism underlying this immediate analgesic effect, specifically, the means through which the physical mechanical stimulus is converted into specific biochemical signals, remains incompletely understood. The key molecular sensors and signal transduction pathways involved have not yet been completely elucidated [22–25]. In the present study, we systematically explored the peripheral initiation mechanism underlying acupuncture-induced analgesia. Our results suggest that acupuncture-generated mechanical stimulation activates the mechanosensitive channel Piezo1 in acupoint myocytes, triggering strong Ca^2^⁺ influx and initiating a downstream Ca^2^⁺-dependent signaling cascade. The elevated intracellular Ca^2^⁺ levels further activate CaM/CaMKII signaling, thereby modulating structural and functional SR–mitochondria coupling and improving mitochondrial energy metabolism. The subsequent increases in the abundance of local ATP and ADO induce peripheral analgesia by activating the A1 receptor. Of note, the ATP–ADO conversion pathway is supported only by correlative changes in metabolite levels; direct evidence for CD39/CD73-mediated enzymatic hydrolysis and discrimination between intracellular and extracellular purinergic signaling is lacking.

The present study results confirmed the efficacy and specificity of the acupuncture intervention. According to behavioral data, acupuncture at ST36 significantly alleviates inflammatory hyperalgesia in mice with inflammatory pain. This is evidenced by an immediate and marked increase in the thermal pain threshold after acupuncture, while shallow needling at the same acupoint produced no significant analgesic effect. This depth-dependent induction of analgesic effect is highly consistent with extensive clinical experience and the findings presented in research reports. This finding indicates that deep needling or deep needling combined with needle manipulation, which often induces the “Deqi sensation,” exhibits higher analgesic efficacy in acute pain management [26–30]. Modern neuroimaging studies, such as fMRI, have provided central-level evidence: deep needling modulates neural activity in sensation-, emotion-, and cognition-related brain regions (e.g., primary/secondary somatosensory cortex, anterior cingulate cortex, insula, and cerebellum) and regulates the functional connectivity network between these regions. By contrast, shallow needling typically only elicits mild skin prickling, evokes a response pattern in the CNS distinct from deep needling, and produces no similar analgesia-related network modulation [31]. More critically, acupuncture-induced changes in local electromyographic activity at the acupoint are closely related to the “Deqi sensation” and analgesic efficacy, and self-contraction of myocytes is considered a crucial initial step in triggering this series of physiological responses [32]. Our study offers direct molecular-level support: deep needling specifically and significantly upregulates the CaM protein expression in the muscle layer tissue of the ST36 acupoint and strongly activates its downstream CaMKII. Although shallow needling can slightly activate CaMKII in the non-muscular connective tissue, it exerts a weak regulatory effect on the CaM/CaMKII axis in the muscle layer. These results collectively indicate that the muscle layer is the core target area for acupuncture-induced analgesia, suggesting that muscular tissue actively participates in the perception and transduction of acupuncture-induced mechanical signals. This acupoint-specific phenomenon is closely related to the distinct neuroanatomical innervation of ST36 and ST25. ST36, located in the hindlimb, projects afferent signals to the lumbar spinal cord (L4–L6), which corresponds to the segmental input from the inflamed paw, thereby enabling segmental analgesia via spinal and descending modulation. By contrast, ST25 projects afferent signals to thoracolumbar segments (T10–L1) that do not overlap with hindlimb afferents, which explains its lack of efficacy. Furthermore, a subset of PROKR2-expressing sensory neurons that specifically innervate deep hindlimb fascia, but not abdominal fascia, has been identified as essential for mediating ST36 EA-induced analgesia, thereby providing a molecular basis for this acupoint specificity [9, 33]. Integrating these findings with our own, we found that acupuncture-generated mechanical force at the ST36 acupoint activates the Piezo1 channel on muscle cells, triggering the CaM/CaMKII signaling cascade. This signaling subsequently promotes local energy metabolic reprogramming and ADO release. This mechanism may function synergistically with the lumbar spinal cord–vagal reflex pathway originating from ST36, collectively contributing to the neurobiological basis underlying the acupoint-specific analgesic effects of acupuncture at ST36.

To further clarify the upstream trigger of acupuncture-initiated Ca^2^⁺ signaling, we focused on the mechanosensitive channel Piezo1. Piezo1 is a membrane tension-sensitive cation channel permeable to Ca^2^⁺ and a critical mechanical sensor in multiple cell types. Our results demonstrated that Piezo1 is abundantly expressed in myocytes at ST36 and exhibits a distribution pattern overlapping with that of CaM, offering morphological evidence for their functional interaction. Gain- and loss-of-function assays further verified the regulatory role of Piezo1. The Piezo1 agonist Yoda1 mimicked acupuncture-induced analgesia and activated CaM/CaMKII signaling, whereas genetic knockdown or pharmacological inhibition of Piezo1 attenuated acupuncture-mediated pain relief and downstream pathway activation. Consistent with these molecular results, in vivo two-photon Ca imaging dynamically captured Ca transients in myocytes at the acupoint during EA stimulation. These Ca responses were substantially reduced by Piezo1 inhibition. Of note, EA cannot fully recapitulate the mechanical characteristics of manual acupuncture. Therefore, relevant Ca signaling findings need to be interpreted with caution. Extracellular Ca^2^⁺ chelation also partially abolished acupuncture-induced analgesia, further confirming that Piezo1-dependent Ca^2^⁺ influx acts as an initial trigger for the downstream signaling cascade induced by acupuncture [34]. Collectively, these findings highlight Piezo1 as a key upstream mechanosensor mediating peripheral signal activation induced by acupuncture. Studies have also reported that Piezo1 is involved in acupuncture-mediated regulation of gastrointestinal and cardiovascular functions [35, 36], implying its broader role in the physiological effects of acupuncture. Our future work will explore whether other mechanosensitive channels, including TRPV4, TRPA1, and TREK-1, cooperate or interact with Piezo1 during needling stimulation. Moreover, graded needling intensities should be adopted in follow-up studies to define the threshold and saturation characteristics of acupuncture-induced analgesia.

CaM, the most versatile and essential Ca sensor in eukaryotic cells, plays a central role in converting Ca^2^⁺ signals into various cellular functions. Upon binding Ca^2^⁺, the conformation of CaM changes, thus enabling it to activate numerous downstream effector proteins, including CaMKII, and forming complex signaling networks [37–40]. Consistent with studies reporting central and spinal CaM/CaMKII modulation during acupuncture-induced analgesia [41–43], our peripheral results further demonstrated that acupuncture activates the CaM/CaMKII axis specifically in acupoint myocytes. IF staining revealed that CaM is widely distributed in multiple local cell types. However, functional validation indicated that myocytic CaM predominantly contributes to acupuncture-induced analgesia. Specific knockdown or pharmacological inhibition of CaM suppressed acupuncture-induced analgesia and CaMKII activation, while artificial elevation of Ca^2^⁺ levels aggravated inflammatory hyperalgesia. These findings suggest that myocyte-derived CaM/CaMKII signaling is necessary for propagating acupuncture-generated mechanical signals, whereas non-muscular cell populations may primarily modulate the local tissue microenvironment. This finding is consistent with the established role of Ca^2^⁺-mediated cellular signaling in pain regulation [44, 45].

Notably, our study reveals a dual-source Ca amplification mechanism underlying acupuncture-induced analgesia. The initial Piezo1-mediated extracellular Ca^2^⁺ influx acts as a triggering signal that promotes SR Ca^2^⁺ release via IP3R activation, thereby amplifying intracellular Ca signaling. Given that Ca^2^⁺ serves as a co-agonist of IP3R, ryanodine receptor (RYR)-mediated Ca-induced Ca release (CICR) is also likely to contribute to this process, which can be verified using RYR-targeted pharmacological tools [46]. This CICR-dependent secondary Ca elevation sustains downstream CaMKII activation. Mechanistically, acupuncture enhances SR–mitochondrial structural coupling and upregulates the IP3R-GRP75-VDAC1-MCU axis, thereby facilitating mitochondrial Ca uptake and energy metabolism. As cytosolic Ca dynamics were assessed in this study, mitochondrial Ca must be directly visualized using specific probes such as Rhod-2 to completely validate Ca transfer between SR and mitochondria [47]. Although we detected increased activity of mitochondrial CaMKII (Fig. 5U), serving as indirect biochemical evidence supporting altered mitochondrial calcium signaling after acupuncture, only cytosolic Ca dynamics were directly assessed in this study. Mitochondrial Ca^2^⁺ dynamics must be directly visualized using specific probes such as Rhod-2 to completely validate the proposed Ca transfer between SR and mitochondria. Consequently, local ATP and ADO levels are elevated after acupuncture-induced mechanical stimulation. Increased ADO levels further activate peripheral A1 receptors to mediate analgesic effects. This finding is supported by DPCPX blockade and CPA rescue experiments. Importantly, Piezo1 knockdown abolished acupuncture-induced SR–mitochondrial remodeling, metabolic elevation, and purinergic signal upregulation, confirming that Piezo1 is a critical upstream regulator of this entire cascade. Taken together, the aforementioned SR–mitochondrial Ca signaling network should be regarded as a working model inferred from multiple indirect experimental observations, pending further verification with more selective pharmacological reagents and direct mitochondrial calcium recording.

Several limitations of this study should be explicitly acknowledged. First, we adopted unified and standardized manual acupuncture protocols throughout the experiments. However, we did not quantitatively measure mechanical parameters such as force magnitude, tissue strain, and torque. The dose–response threshold and saturation effects of different needling intensities were also not systematically explored. Second, all behavioral assessments were conducted exclusively in a CFA-induced inflammatory pain model of male mice only. Female mice were excluded to avoid cyclic estrogen change-induced fluctuations in pain sensitivity, a common practice in pain research. This experimental design may reduce the generalizability of our findings to other pain phenotypes and female subjects. Third, we focused on myocyte-specific Piezo1 function via viral knockdown; however, Piezo1 is also expressed in mast cells, fibroblasts, and endothelial cells within acupoint tissues. The auxiliary contributions of these non-myocyte cell populations cannot be completely ignored. Fourth, local microinjection of a 20 μL solution at ST36 may induce mild tissue irritation resulting from needle puncture, representing a potential confounding factor. Additionally, baseline tests confirmed that knockdown of Piezo1 or CaM alone caused no alterations in basal pain thresholds, partially excluding the intrinsic effects of genetic manipulation on nociception. Fifth, it should be noted that the pharmacological tools applied in the present study possess certain documented off-target activities. For instance, the IP3R blocker 2-APB exerts broad non-selective inhibitory effects on diverse ion channels [48]. We did not perform parallel experiments using the more selective IP3R antagonist xestospongin C, owing to technical challenges in obtaining stable in vivo experimental outcomes. GsMTx4 exerts broad inhibitory effects on Piezo1, Piezo2 and multiple TRP channels via modulating lipid bilayer tension. Although Yoda1 preferentially targets Piezo1, recent neuronal data shows it elicits mild Piezo2-dependent calcium signals in sensory neurons. To unambiguously separate the respective functions of Piezo1 and Piezo2 in acupoint mechanotransduction, future investigations could employ highly Piezo1-selective antagonists including Dooku1 combined with cell-type-specific genetic labeling strategies [49–51]. Moreover, we have not systematically examined the functional contribution of RYR channels in the present work, which requires dedicated pharmacological testing in follow-up studies. Sixth, as mentioned above, direct evidence for CD39/CD73-dependent ATP–ADO conversion and differential intracellular/extracellular purinergic signaling is not available; the downstream ATP–ADO–A1R axis is therefore interpreted as a correlative working model rather than a fully validated causal pathway. Seventh, Ca imaging data were acquired following EA stimulation, which differs from manual acupuncture in its mechanical stimulation patterns. Therefore, caution should be exercised when extrapolating these mechanistic findings to manual acupuncture. Finally, the present study’s findings are derived from animal experiments. As interspecies differences exist in acupoint anatomy and pain regulation, the clinical translational value needs to be validated in human studies.

In conclusion, the present study delineates a plausible peripheral mechanotransduction pathway for acupuncture-induced analgesic effects specific to CFA-induced inflammatory pain. Acupuncture-generated mechanical stimuli activate myocytic Piezo1 to induce initial Ca^2^⁺ influx, which activates the CaM/CaMKII cascade. This cascade further enhances SR–mitochondrial coupling, promotes mitochondrial energy metabolism, and increases the abundance of local ATP and ADO. The accumulated ADO subsequently activates peripheral A1 receptors to alleviate inflammatory pain. All findings in this study are derived from the inflammatory pain model in male mice. These results offer a peripheral molecular explanation for the mechanisms underlying acupuncture-induced mechanical signal transduction and purinergic analgesic modulation, advancing our mechanistic understanding of acupoint-specific pathways that mediate acupuncture-induced analgesia.

## Supplementary Information

Additional file 1.

## Data Availability

The data that support the findings of this study are available from the corresponding author upon reasonable request.

## Abbreviations

2-APB: 2-Aminoethoxydiphenyl borate
5-HT1A: 5-Hydroxytryptamine 1A
AAV: Adeno-associated Virus
ADO: Adenosine
ATP: Adenosine Triphosphate
BDNF: Brain-Derived Neurotrophic Factor
CaM: Calmodulin
CaMKII: Ca^2+^/Calmodulin-dependent protein Kinase II
cAMP: Cyclic Adenosine Monophosphate
CFA: Complete Freund’s Adjuvant
CICR: Calcium-Induced Calcium Release
CREB: CAMP Response Element-Binding Protein
EA: Electroacupuncture
ELISA: Enzyme-Linked Immunosorbent Assay
EMG: Electromyography
GRP75: Glucose-Regulated Protein 75
iEMG: Integrated Electromyography
IF: Immunofluorescence
IHC: Immunohistochemistry
IP3R: Inositol 1,4,5-Trisphosphate Receptor
MAMs: Mitochondria-Associated Endoplasmic Reticulum Membranes
MCU: Mitochondrial Calcium Uniporter
PKC: Protein Kinase C
PWL: Paw Withdrawal Latency
shRNA: Short Hairpin RNA
SR: Sarcoplasmic Reticulum
TEM: Transmission Electron Microscopy
TFP: Trifluoperazine
TRPA1: Transient Receptor Potential Ankyrin 1
TRPV1: Transient Receptor Potential Vanilloid 1
TRPV4: Transient Receptor Potential Vanilloid 4
TREK-1: TWIK-related K^+^ Channel 1
VDAC1: Voltage-Dependent Anion Channel 1
WB: Western Blot
