# Supplementary material for: Piezo1-mitochondrial calcium uniporter coupling-dependent mechanotransduction basis for acupuncture to drive analgesia
Source: Chin Med. 2026 Oct 28;21:231. doi: 10.1186/s13020-026-01502-0 (PMC13715242; doi:10.1186/s13020-026-01502-0)
Supplement: Supplementary file 1 [file 13020_2026_1502_MOESM1_ESM.docx]

**Supplement file 1**

**Supplement Figures**

Fig. 3 Deep acupuncture at ST36 alleviated pain by upregulating the CaM expression and CaMKII phosphorylation in the muscle layer. (A) Schematic illustration of the experimental schedule (Day -1: baseline; Day 0: CFA immunization for AA model induction; Day 1: sample collection 2-h post-acupuncture intervention). (B) PWL to radiant heat in different groups. Data are presented as the mean ±SEM, as analyzed by one-way ANOVA, followed by post-hoc test (^**^P < 0.01 vs. baseline; ^##^P < 0.01 vs. CFA group; n = 6). (C) PWL to radiant heat in different groups. Data are presented as the mean ±SEM, as analyzed by one-way ANOVA, followed by post-hoc test (^**^P < 0.01 vs. baseline; ^##^P < 0.01 vs. CFA group; n = 6). (D–F) WB analysis of p-CaMKII/CaMKII and p-CaMKII/GAPDH ratios in the non-muscle layer. Data are presented as the mean ±SEM, as analyzed by one-way ANOVA, followed by post-hoc test (^*^P < 0.05, ^**^P < 0.01 vs. Con group; ^#^P < 0.05, ^##^P < 0.01 vs. CFA group; n = 6). (G–I) WB analysis of p-CaMKII/CaMKII and p-CaMKII/GAPDH ratios in the muscle layer. Data are presented as the mean ±SEM, as analyzed by one-way ANOVA, followed by post-hoc test (^**^P < 0.01 vs. Con group; ^#^P < 0.05, ^##^P < 0.01 vs. CFA group; n = 6). (J) IHC staining and quantification of CaM in the non-muscle layer. Data are presented as the mean ±SEM, as analyzed by one-way ANOVA, followed by post-hoc test (^*^P < 0.05, ^**^P < 0.01 vs. Con group; ^#^P < 0.05, ^##^P < 0.01 vs. CFA group; Scale bar = 100 µm; n = 6). (K) IHC staining and quantification of CaM in the muscle layer. Data are presented as the mean ±SEM, as analyzed by one-way ANOVA, followed by post-hoc test (^*^P < 0.05 vs. Con group; ^##^P < 0.01 vs. CFA group; ^&^P < 0.05 vs. ACU group; Scale bar = 100 µm; n = 6). SACU: shallow acupuncture; ACU: deep acupuncture.
